# Breeding for resilience in finishing pigs can decrease tail biting, lameness and mortality

**DOI:** 10.1186/s12711-024-00919-1

**Published:** 2024-06-20

**Authors:** Wim Gorssen, Carmen Winters, Roel Meyermans, Léa Chapard, Katrijn Hooyberghs, Jürgen Depuydt, Steven Janssens, Han Mulder, Nadine Buys

**Affiliations:** 1 Center for Animal Breeding and Genetics, Department of Biosystems, KU Leuven, Kasteelpark Arenberg 30, Box 2472, 3001 Leuven, Belgium; 2 Vlaamse Piétrain Fokkerij Vzw, Aardenburgkalseide 254, 9990 Maldegem, Belgium; 3 Wageningen University & Research Animal Breeding and Genomics, P.O. Box 338, 6700 AH Wageningen, the Netherlands; 4 Animal Physiology, Institute of Agricultural Sciences, ETH Zurich, 8092 Zürich, Switzerland

## Abstract

**Background:**

Previous research showed that deviations in longitudinal data are heritable and can be used as a proxy for pigs’ general resilience. However, only a few studies investigated the relationship between these resilience traits and other traits related to resilience and welfare. Therefore, this study investigated the relationship between resilience traits derived from deviations in longitudinal data and traits related to animal resilience, health and welfare, such as tail and ear biting wounds, lameness and mortality.

**Results:**

In our experiment, 1919 finishing pigs with known pedigree (133 Piétrain sires and 266 crossbred dams) were weighed every 2 weeks and scored for physical abnormalities, such as lameness and ear and tail biting wounds (17,066 records). Resilience was assessed via deviations in body weight, deviations in weighing order and deviations in observed activity during weighing. The association between these resilience traits and physical abnormality traits was investigated and genetic parameters were estimated. Deviations in body weight had moderate heritability estimates (h^2^ = 25.2 to 36.3%), whereas deviations in weighing order (h^2^ = 4.2%) and deviations in activity during weighing (h^2^ = 12.0%) had low heritability estimates. Moreover, deviations in body weight were positively associated and genetically correlated with tail biting wounds (r_g_ = 0.22 to 0.30), lameness (r_g_ = 0.15 to 0.31) and mortality (r_g_ = 0.19 to 0.33). These results indicate that events of tail biting, lameness and mortality are associated with deviations in pigs’ body weight evolution. This relationship was not found for deviations in weighing order and activity during weighing. Furthermore, individual body weight deviations were positively correlated with uniformity at the pen level, providing evidence that breeding for these resilience traits might increase both pigs’ resilience and within-family uniformity.

**Conclusions:**

In summary, our findings show that breeding for resilience traits based on deviations in longitudinal weight data can decrease pigs’ tail biting wounds, lameness and mortality while improving uniformity at the pen level. These findings are valuable for pig breeders, as they offer evidence that these resilience traits are an indication of animals’ general health, welfare and resilience. Moreover, these results will stimulate the quantification of resilience via longitudinal body weights in other species.

**Supplementary Information:**

The online version contains supplementary material available at 10.1186/s12711-024-00919-1.

## Background

Resilience is a multifaceted concept overlapping with other related concepts such as robustness, tolerance, and resistance [1]. In this study, we define resilience based on Berghof et al. [2], building on Colditz and Hine [3], as “the capacity of the animal to be minimally affected by disturbances or to rapidly return to the state pertained before exposure to a disturbance”. This definition implies that disturbances can affect animals, albeit with individual variation in the magnitude of the impact exerted by these disturbances. As a result, deviations in longitudinal data can be used as a proxy for overall resilience as more resilient animals will show less deviations from their optimal production level with a quick recovery, after a challenge [4]. Testing this hypothesis of resilience necessitates specific conditions and assumptions. First, the animals tested must encounter at least one challenge since evaluating resilience in an ‘optimal’ environment is unfeasible. Second, collection of longitudinal data is needed, as it is impossible to quantify temporal variability within an individual from a single data point [2]. These longitudinal data may encompass various aspects such as (re)production traits (e.g., weight or feed intake), immunological traits (e.g., viral load, antibody levels), or physiological traits (e.g., body temperature, heart rate) [2]. However, to quantify resilience effectively, these traits must be impacted by the challenges under consideration. And third, inferring the optimal (expected) performance of an animal from longitudinal data necessitates proper statistical modeling. Only then can deviations from this “optimal” performance be quantified.

Breeding resilient livestock is becoming increasingly important [4, 5]. The increasing intensification in pig farming, especially in the European Union (EU), results in more pigs to be managed per farmer [6]. As a result, improving pigs’ resilience is desired as it would reduce costs of labor and treatment while enhancing animal welfare and maintaining optimal production levels [5, 7, 8]. In addition, the goal of the EU Green Deals’ farm to fork strategy is to reduce antimicrobials by 50% in livestock and aquaculture by 2030 [9]. Besides, the focus on animal welfare is increasing in the EU, framed by a stringent welfare legislation. A recent example is the European citizens’ initiative ‘End the cage age’ [10], which could potentially lead to the prohibition of all cage systems in pig husbandry. The call to raise pigs in a more natural setting implies exposing them to a broader spectrum of challenging environments. In addition, the escalating impact of climate change is resulting in a significant rise in the frequency of heat waves [11, 12]. Finally, there is mounting pressure to incorporate more by-products into pig feed to alleviate food-feed competition. However, augmenting the proportion of by-products in pigs’ diets may present a dietary challenge, certainly since most breeding pigs are selected on high-quality, nutrient rich diets [13].

Scheffer et al. [4] indicated that a holistic approach might be optimal for improving general resilience. Indeed, previous studies have shown that breeding against specific diseases can be effective, but might increase susceptibility to other diseases [1, 14] and as such, not improve general resilience. Moreover, resilience indicators based on deviations from longitudinal data are more informative, as they reflect more continuous variation in phenotypical differences compared to classical scoring of resilience indicators, such as mortality (”dead” vs “alive”) or disease status (”not”, ”mildly” or “severely” affected) [15, 16]. For these reasons, Scheffer et al. [4] suggested that these new resilience traits based on deviations in longitudinal data could have substantial impact.

In the past, collecting longitudinal data was often practically challenging and expensive. However, due to technological developments the collection of longitudinal data will probably become standard in animal breeding [4]. Automatic feeding stations can already record individual pigs’ body weight, feed intake and feeding behaviour at every visit. Moreover, computer vision and wearable devices can phenotype pigs almost continuously [4], for example to monitor body composition [17], tail biting events [18] heart rate, respiration rate and body temperature [19].

Previous research showed that deviations in longitudinal data are heritable and these studies suggested that these resilience traits can be used as a proxy for pigs’ general resilience. Deviations in daily feed intake were estimated to be moderately heritable, with heritability (h^2^) estimates ranging from 8 to 26% [20], 7 to 11% [21], 9 to 23% [22]. Deviations in feeding behaviour, such as number of daily visits or daily visit duration, generally had higher heritabilities ranging from 16 to 20% [21], 16 to 28% [22] and 36 to 40% [23]. Heritability estimates of pigs’ body weight deviations range from 3 to 4% [24], 3 to 20% [22] and 31% [25], depending on the study. However, only a few studies looked at the relationship of these “indirect” traits with specific resilience-related traits in pigs, such as disease, mortality and social stressors. Putz et al. [20] and Cheng et al. [26] estimated moderate to high genetic correlations (r_g_ = 0.37 to 0.85) between deviations in daily feed intake and mortality and antibiotic treatments, indicating that more deviations were associated with an increased mortality and an increased number of antibiotic treatments. In another study, a phenotypical association was found between deviations in activity levels and resilience in pigs infected with porcine reproductive and respiratory syndrome virus (PRRSV) [27]. Here, the odds of being non-resilient increased by 1.42 with a one-unit difference in root mean squared error (RMSE) of activity three days post-challenge.

Relationships between traits related to resilience and welfare, on the one hand, and deviations in longitudinal data, on the other hand, have already been observed in other species. In chicken, body weight deviations were positively related with lesion scores after infection [28]. In dairy cattle, a decrease in milk yield variations has been shown to be associated with improved longevity (r_g_ = − 0.28 to − 0.34), udder health (r_g_ = − 0.22 to − 0.32) and ketosis (r_g_ = − 0.27 to − 0.33) [29]. In humans, general or systemic resilience based on deviations from natural time series, such as recovery time of blood pressure or heart rate, are related with overall mortality risk [4].

In the current study, resilience traits were estimated as deviations in body weight, deviations in weighing order and deviations in activity levels during weighing. First, resilience traits from deviations in longitudinal data were associated with physical abnormality scores and mortality. Second, variance components and heritabilities were estimated for these traits. And third, genetic correlations were estimated between resilience traits and traits related to resilience and welfare (e.g. mortality and tail biting) to evaluate if deviation traits are informative for breeders to improve pigs’ general resilience.

## Methods

### Animals and data collection

In total before quality control, 3696 finishing pigs with 20,833 body weight recordings from Vlaamse Piétrain Fokkerij (Belgium) were examined in this study. All pigs were crossbreds originating from Piétrain sires (N = 141) and Pig Improvement Company (PIC)-based dams bred in a criss-cross system (N = 352) with known pedigree. Piétrain sires were not preselected for this study and were a representative sample for the population. Most Piétrain sires from this study were homozygous positive for the halothane sensitivity (*RYR1*) genotype (93.3%), while 4.2% of the boars were heterozygous and 2.5% of the boars were homozygous negative.

Before weaning, piglets were vaccinated at the sow farm against *Mycoplasma hyopneumoniae*, as routinely done in Belgian pig production. At a mean age of 73.3 days (sd = 3.0) and weight of 23.2 kg (sd = 4.5), pigs were transferred from the sow farm to the experimental farm and upon arrival, pigs received anthelminthic medication and vaccination against *Lawsonia intracellularis*. Pigs were raised between July 2020 and July 2021 in a single pig building with 17 identical compartments composed of eight fully slatted pens (2.5 m × 4.0 m) per compartment. New pigs entered the stable in batches of 200 to 320 pigs every 2 weeks, filling two or three compartments with the progeny of 8 to 12 Piétrain sires. Per sire, a median of 26 crossbred piglets (half-sibs and full-sibs from the same sire) were equally distributed over two mixed-sex pens (sows and barrows) in the same compartment, mostly 13 piglets per pen. Once the stable was filled, it took about 2 months before a new round of pigs could enter, as the previous pigs were not sent to slaughter yet. During this experiment, we were able to evaluate 17 batches of pigs, distributed over 317 pens. Food and water were available to the animals ad libitum and were supplied via one trough and one nipple drinker per pen throughout the duration of the experiment. Over the course of the finishing period, which lasted about 120 days, observations were made until the pigs reached slaughter weight. The mean slaughter age and weight were 191.0 days (sd = 25.1) and 121.8 kg (sd = 10.4). The specific challenges presented to the pigs in our experiment were not recorded. However, pigs were kept in commercial conditions, automatically creating at least some challenges of various origin over the finishing period. Examples of such challenges are transportation stress upon arrival, disease pressure, heat stress in summer, social stress and stress related to a feed transition halfway the finishing phase.

#### Data collection

The data collection and experimental setup used in this study was previously described in detail by Gorssen et al. [30]. In short, pigs were weighed individually using radio frequency identification (RFID) ear tags and a RFID reader connected to a ground scale weighing platform. In total, 3358 pigs were weighed three days and 13 days after arrival on the farm. Hereafter, due to practical limitations, a subset of one out of two pens per sire was selected for subsequent weight recordings, at least at bi-weekly intervals (1919 pigs with 17,066 body weight recordings). For all pigs, slaughter weight and lean meat percentage were individually collected at the slaughterhouse of the Belgian Pork Group (site Meer, Belgium) using AutoFom III™ (Frontmatec, Smoerum A/S, Denmark) [31]. Moreover, cumulative feed intake was recorded weekly at the pen level for all pens. As explained by Gorssen et al. [32], weighing order on the pen level was recorded per weighing. Within a pen, the pig that was weighed first was scored with ‘1’, the second with ‘2’, etc.

In addition, during each weighing, pigs were visually scored individually on a set of physical abnormalities by the first author: presence of umbilical hernia (0 = not present, 1 = present), ear swellings or hematomas (0 = none, 1 = one ear, 2 = both ears), lameness (0 = not lame, 1 = lame), ear biting wounds (0 = none, 1 = one ear, 2 = both ears) and tail biting wounds (0 = none, 1 = small scratches, 3 = bloody and/or infected tail; (see Additional file 1 Figure S1). These scores were treated as continuous variables in further analyses. For tail biting wounds, we choose to not score bloody/infected tails with a score of ‘2’ but with a score of ‘3’, to give more weight to severely wounded tails. We would like to note that the used scoring method for these physical abnormalities is imperfect, as we assume that these scores are equidistant and can be treated as continuous variables. Although these measurements are imperfect and partly subjective, they will at least partly be related to pigs’ health, welfare and/or resilience status. Furthermore, although medical treatments were often started upon onset of problems, these treatments were not recorded in this study.

Estimates of activity levels during weighing using video analysis were only available for a subset of the total dataset and were recorded between January 2021 and July 2022 (1556 pigs, 7428 records), as described in Gorssen et al. [30]. In short, the activity level was estimated based on a linear combination of the mean speed, straightness index and sinuosity index of a pig’s tail base coordinates over time during weighing, with a 1/3 weight per trait after rescaling [31].

Ear tissue was collected for at least one finishing pig per sire-dam combination using the ‘Tissue Sampling Unit for DNA testing’ from Allflex® (New Zealand). In total, 413 finishing pigs were genotyped on medium-density single nucleotide polymorphism (SNP) chips: 122 finishing pigs were genotyped on the IMAGE porcine array (10,107 SNPs), whereas 291 pigs were genotyped on the GGP Porcine 50K chip from Neogen® (USA). Two SNP genotyping arrays were used, because these pigs were genotyped in different projects. Furthermore, the Piétrain sires were previously genotyped on the GGP Porcine 50K chip and the data were available for this research. Collection of ear tissues were approved by the Animal Ethics Committee of KU Leuven (P004/2020).

### Data handling and quality control of weight records and genotypes

Before quality control (QC), 20,833 individual body weight records were available on 3696 pigs from 317 pens. First, we checked for duplicated records (same ID appearing in different pens or multiple recordings on the same day) and we removed duplicates if needed. Next, the number of unique sires per pen was checked and pens with more than one unique sire were removed (N = 1 pen, 24 records). Outlying weights were detected for pigs with at least four weight records using second order polynomial regression and comparing predicted with observed weights. Based on the histogram of deviations from predicted vs observed weights, deviations of more than three standard deviations were visually inspected. However, all weight trajectories seemed valid and no outlying weights were set to missing.

Genotype QC was done with the PLINK v1.9 software [33] (PLINK commands in brackets). The same QC settings were used for both arrays. Individual QC was first done on outlying missing (–missing) and heterozygosity (–het) values, with a minimum individual call-rate of 85% and heterozygosity maximum three standard deviations from the mean. Eight pigs did not pass this QC step due to 15 to 24% missing SNPs. Hereafter, QC of SNPs was performed by retaining only autosomal SNPs (–autosome) and setting the SNP call rate at a minimum of 95% (–geno 0.05). Lastly, duplications were checked (–genome), but all samples had relatedness (PI_HAT) < 0.90.

Finishing pigs genotyped on the IMAGE array [34] had 9072 SNPs and 122 pigs were kept after QC whereas for the finishing pigs genotyped on the GGP porcine array 45,824 SNPs and 283 pigs remained. After QC, arrays were merged (–bmerge) resulting in a dataset of 3769 overlapping SNPs and 405 finishing pigs. This dataset was subsequently merged with genotypes from Piétrain sire line (100% overlap, 2805 genotypes after merging) for parentage verification using the seekparentf90 software from the *blupf90* suite of programs [35]. Parentage was not confirmed for three pigs. Although parentage was confirmed for their pedigree (half)sibs, it was decided to remove the complete pens with uncertain paternity (N = 3 pens, 313 records).

**Fig. 1:**
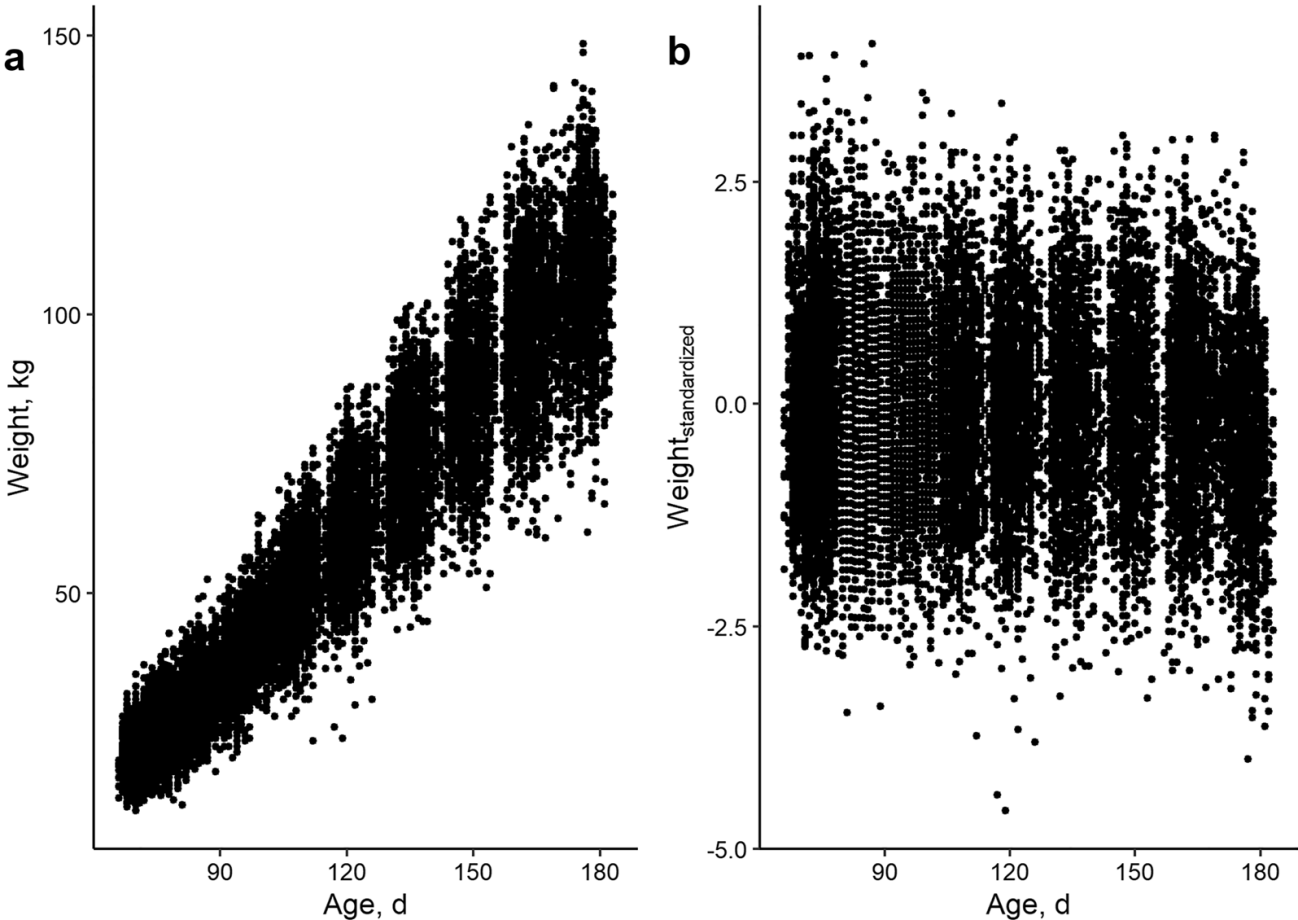
Evolution of weight according to age in finishing pigs. **a** Evolution of weight (kg) according to age (days) for all weight records (17,066 records on 1919 pigs), excluding slaughter weights and **b** Transformation of weights to standard normal distribution with a mean of 0 and a standard deviation of 1

After QC for both phenotypic records and genotypes, the dataset contained 20,496 records on 3649 pigs (Fig. 1a). Resilience traits were constructed based on weight deviations, which were only estimated for pigs with at least five records. Consequently, a dataset of 17,066 records on 1919 pigs were left for analysing longitudinal resilience traits. These pigs, housed in 151 pens on paternal full- and half-sib basis, descended from 133 sires and 266 dams.

### Trait definition

After QC, new traits were defined at the individual pig level. First, average daily gain (ADG) was determined as weight gain (in kg) divided by period of time in days. ADG from birth until first weight recording upon entering the finishing farm was named ADG_youth_, whereas ADG from birth until last weight recording was named ADG_life_. As birth weights were not recorded, weight at first/last recording was simply divided by age in days to calculate ADG_youth_ and ADG_life_. For physical abnormalities, scored at each weighing event, the maximum score per pig was retained. For example, if a pig had severe tail biting wounds (score ‘3’), but this wound healed over time (score ‘0’ or ‘1’), the maximum score of ‘3’ was retained for statistical analysis. The sum of scores per individual was also calculated to estimate the duration of an event. However, the (genetic) correlations with a maximum score were very high. Therefore, only the results for maximum score are shown in this paper (detailed results not shown). Average feed intake (FI; kg/d) and feed conversion ratio (FCR; kg/kg) were estimated at the pen level. FI was estimated as the total amount of feed intake of the pen in kg, divided by the number of pigs and duration in days. FCR was estimated by dividing total feed intake (kg) by cumulative weight of a pen (kg), including pigs that died. Moreover, the coefficients of variation (CV) of pigs’ weight within a pen were estimated for weighing of pigs between 70 and 89 days of age (CV_start_; mean age of 81.8 days, sd = 3.7), weighings between 115 and 130 days of age (CV_middle_; mean age of 120.8 days, sd = 3.0), and of weighings between 170 and 185 days of age (CV_end_; mean age of 176.2 days, sd = 2.8), as $\text{CV}=\frac{\text{Standard deviation of weight at pen level}}{\text{Mean weight at pen level}}$.

**Fig. 2:**
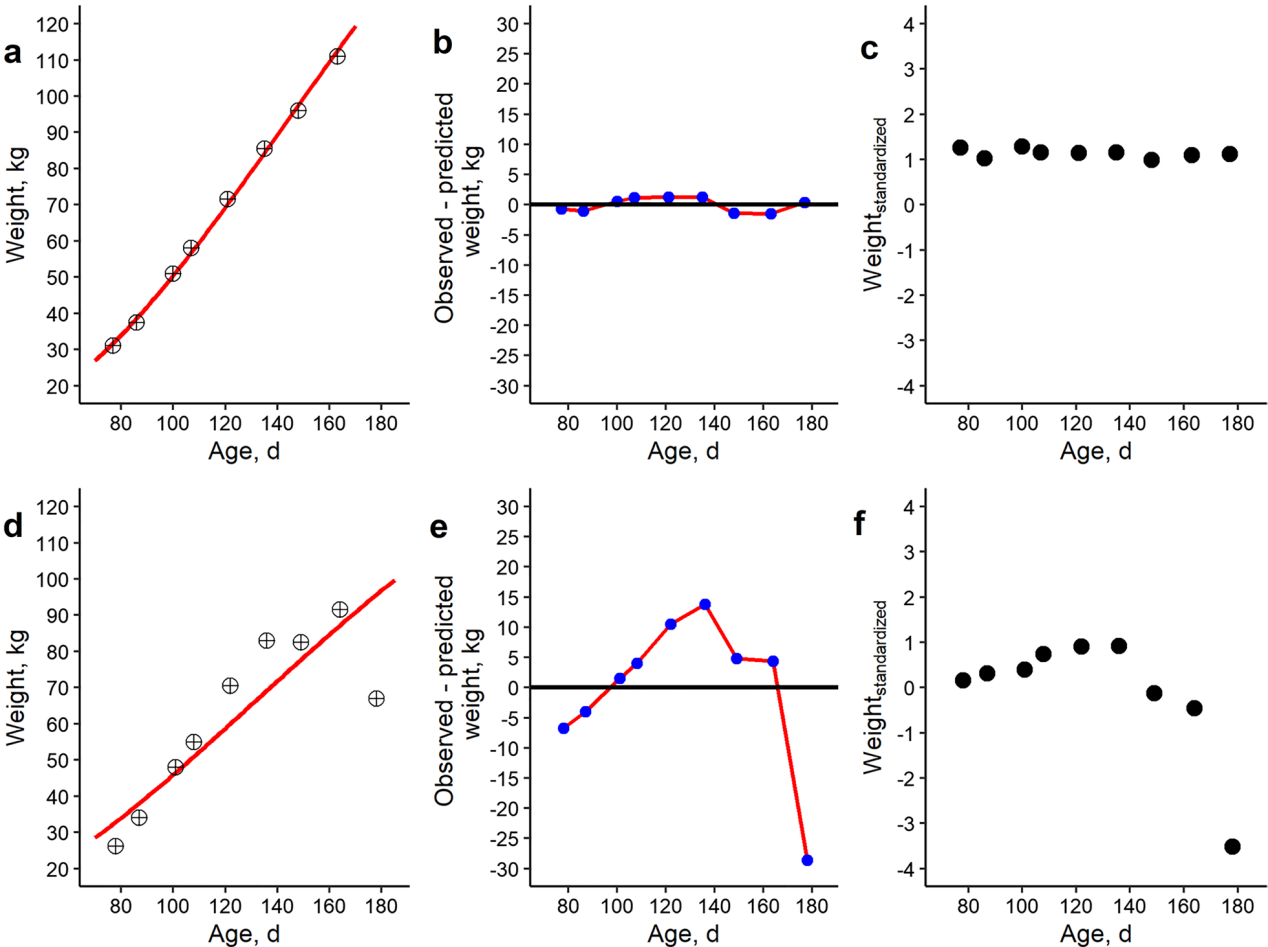
Weight evolution in trait units and after standardization, Gompertz estimates and deviation from Gompertz for two pigs. **a**, **d** Show observed weights in kg (dots) with the Gompertz growth curve as a solid red line. **b**, **e** Show deviation in kg from observed weights vs Gompertz predicted weights. **c**, **f** Show evolution of standardized weight by age bin of 3 days. Images **a–c** show a pig with almost no deviations between predicted and observed weights, and an above average weight (± 1 sd above population average). Images **d–f** show an individual with strong deviations, especially after 140 days of age. For this pig, severe tail wounds (score 3) were observed from 149 days of age onwards, which resulted in lameness and euthanization at an age of 178 days. This pig had an average to above average weight up to this point but lost a lot of weight afterwards

Resilience traits were constructed based on deviations in longitudinal weight data. Theoretical or predicted weights were estimated using linear modelling and Gompertz growth curve modelling [36] in R [37]. As done by [9], a linear regression of weight as a function of age was performed (*lmer* function in R) and then the root mean squared error (RMSE) was estimated of observed versus predicted weights. This approach seems justified, as finishing pigs are more or less in their linear growth phase [38]. Then, we transformed RMSE using the natural logarithm (*ln* function in R) of the MSE (lnMSE_weight_). Gompertz modelling was performed with the *nls* function and the formula:

$${\text{weight}}_{\text{ij}}={\text{A}}_{\text{i}}*{\text{e}}^{-{\text{B}}_{\text{i}}*{\text{e}}^{{\text{k}}_{\text{i}}*{\text{t}}_{\text{ij}}}}+{\upvarepsilon }_{\text{ij}},$$

where the weight of an individual $\text{i}$ at a given age in days $\text{j}$ (weight_ij_) can be predicted based on a combination of three Gompertz growth curve parameters (${\text{A}}_{\text{i}}$, ${\text{B}}_{\text{i}}$ and ${\text{k}}_{\text{i}}$) with a given time or age ${\text{t}}_{\text{ij}}$, whereas ${\upvarepsilon }_{\text{ij}}$ is residual error. Per individual and per weighing, this approach led to an observed weight and a predicted Gompertz weight (Fig. 2a and d). Hereafter, we calculated the difference between observed and predicted weights (Fig. 2b and e). Finally, the natural logarithm of the variance of these differences in observed versus expected weights was estimated (lnvar_weight_) according to [2]. Next, we also calculated the natural logarithm of the variance of a pigs’ weight after standardizing (lnvar_standardized_), as explained in [28]. All weights were standardized by age with a bin of 3 days (for example, ages 75–77, 78–80,…), to ensure a sufficiently large population per age group. After standardization, weights were distributed with a mean of zero and a standard deviation of 1 per age bin (Fig. 1b). Weight standardization might be needed to correct for a scaling effect: as the mean weight increases over time in finishing pigs, the variance tends to increase as well [39]. Figure 2 shows the methodology for estimation of lnvar_weight_ and lnvar_standardized_ for a pig with little deviations in body weight (Fig. 2a–c) and a pig with strong deviations in body weight (Fig. 2d–f).

Finally, deviations in pigs’ weighing order (lnMSE_order_) and deviations in pigs’ mean activity levels during weighing (lnMSE_activity_) were estimated similar to lnMSE_weight_. These traits were linearly regressed to age, and then the natural logarithm of MSE of observed versus predicted values was estimated. However, we were only able to record lnMSE_order_ and lnMSE_activity_ for a subset of 898 pigs because video recordings started halfway through our experiment, as explained in Gorssen et al. [30].

### Statistical analysis of resilience traits and resilience-related traits

**Fig. 3:**
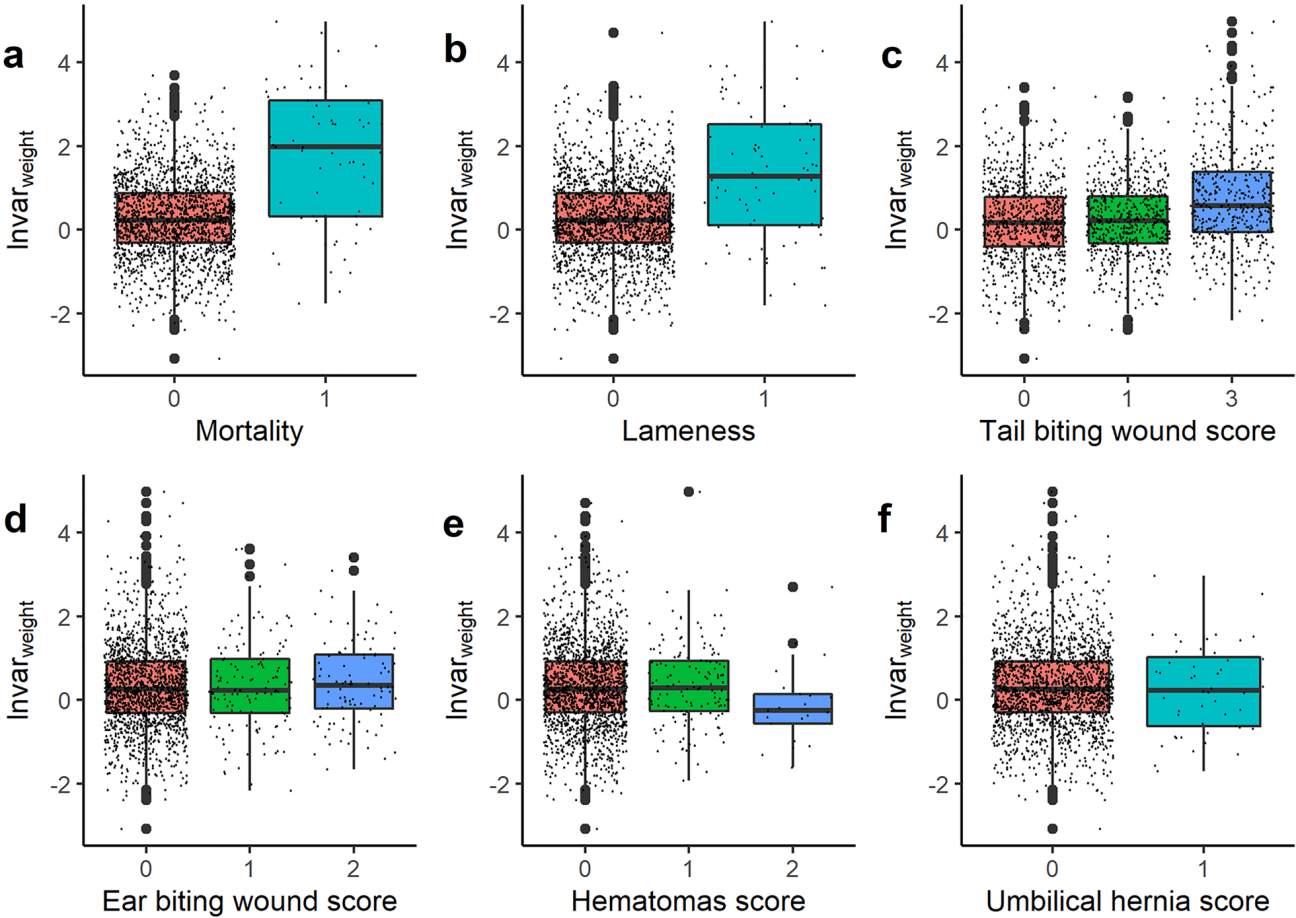
Boxplots of lnvar_weight_ according to mortality and physical abnormality scores. **a** Boxplot of lnvar_weight_ per mortality score. **b** Boxplot of lnvar_weight_ per lameness score. **c** Boxplot of lnvar_weight_ per tail biting wound score. **d** Boxplot of lnvar_weight_ per ear biting wound score. **e** Boxplot of lnvar_weight_ per hematomas score. **f** Boxplot of lnvar_weight_ per umbilical hernia score. Small dots indicate scores for individual pigs. Boxplots for the other resilience traits are also constructed (see Additional file 2 Figure S2, Additional file 3 Figure S3, Additional file 4 Figure S4 and Additional file 5 Figure S5). Effect sizes and significance of these differences were statistically tested and are in Table 1

The statistical associations between the proposed resilience traits lnvar_weight_, lnvar_standardized_, lnMSE_weight_ and lnMSE_activity_ and resilience-related traits, such as mortality and tail and ear biting wounds were investigated. In first instance, boxplots and pairwise correlation plots were visually inspected for resilience traits according to the scores for resilience-related traits (Fig. 3) (see Additional file 2 Figure S2, Additional file 3 Figure S3, Additional file 4 Figure S4, Additional file 5 Figure S5, Additional file 6 Figure S6 and Additional file 7 Figure S7). Hereafter, linear mixed model regression analysis was applied (*lmer* package in R) as follows:

$$\mathbf{y}=\mathbf{X}\mathbf{b}+\mathbf{W}\mathbf{c}+\mathbf{e},$$

where, $\mathbf{y}$ is the vector of phenotypes for a specific resilience trait (lnvar_weight_, lnvar_standardized_, lnMSE_weight_ or lnMSE_activity_); $\mathbf{b}$ is a vector of the fixed effects (sex, 2 levels) and covariables (specific resilience related trait score (tail wounds, ear wounds, hematomas, umbilical hernia, lameness, mortality); age at last recording and ADG_life_); $\mathbf{c}$ is the vector of common environmental pen effects (151 levels), following a normal distribution $\mathbf{c}\sim N(\boldsymbol{0},{\mathbf{I}\upsigma }_{\text{c}}^{2})$, where $\mathbf{I}$ is the identity matrix; $\mathbf{e}$ is the vector of residual effects assumed to follow a normal distribution $\mathbf{e}\sim N(\boldsymbol{0},{\mathbf{I}\upsigma }_{\text{e}}^{2})$; $\mathbf{X}$ and $\mathbf{W}$ are incidence matrices for, respectively, fixed effects, and the random pen effects (including both animal effects and common environmental effects).

### Genetic modelling

Genetic parameters were estimated with the *remlf90* software [35] using single-step genomic prediction (OPTION SNP_file). Heritability was calculated as the ratio of additive genetic variance to total variance, and common environmental pen effect (c^2^) as the ratio of variance explained by common environmental pen effects (c) to total variance. As described by [23, 40, 41] for an exponential model, the genetic coefficient of variation (GCV) was estimated for lnvar_weight_, lnvar_standardized_, lnMSE_weight_ and lnMSE_activity_ as: $\text{GCV}=\sqrt{{\upsigma }_{\text{a}}^{2}}$. Next, we estimated genetic correlations (r_g_) via bivariate animal models for all trait combinations. The estimated animal models were as follows:

$$\mathbf{y}=\mathbf{X}\mathbf{b}+\mathbf{Z}\mathbf{a}+\mathbf{W}\mathbf{c}+\mathbf{e},$$

where, $\mathbf{y}$ is the vector with phenotypes; $\mathbf{b}$ is a vector with the fixed effects (sex, 2 levels; parity, 5 levels) and covariates (maximum age, mean time between consecutive records); $\mathbf{a}$ is a vector of the additive genetic effects (5037 animals in pedigree, 2709 with genotype information). The assumption is that $\mathbf{a}$ follows a normal distribution for the $\mathbf{H}$ matrix following [42–44], using single-step genomic evaluation with both pedigree ($\mathbf{A}$) and genomic ($\mathbf{G}$) relationship matrices: $\mathbf{a}\sim \text{N}(\boldsymbol{0},{\mathbf{H}\upsigma }_{\text{a}}^{2})$. $\mathbf{c}$ is the vector of common environmental pen effects (151 levels), following a normal distribution $\mathbf{c}\sim N(\boldsymbol{0},{\mathbf{I}\upsigma }_{\text{c}}^{2})$, where $\mathbf{I}$ is the identity matrix; $\mathbf{e}$ is the vector of residual effects assumed to follow a normal distribution $\mathbf{e}\sim N(\boldsymbol{0},{\mathbf{I}\upsigma }_{\text{e}}^{2})$; $\mathbf{X}$, $\mathbf{Z}$ and $\mathbf{W}$ are incidence matrices for, respectively, fixed effects, random animal effects and random common environmental pen effects. Within our experimental design, family groups are confounded with pens. The dataset contained 151 pens in total with an average of 12.7 pigs per pen (sd = 1.0; minimum 9.0; maximum 15.0) and every pen was sired by the same sire. Of these 151 pens, 7 pens consisted of full-sibs only, 118 pens consisted of a mixture of full-sibs and half-sibs originating from 2 dams per pen and 26 pens consisted of a mixture of full-sibs and half-sibs originating from 3 dams per pen. The average genetic relatedness across individuals within a single pen was 42.9% (sd = 10.8%; estimated from **H**-matrix). Within the complete set of 1919 studied pigs, the average genetic relatedness was 7.7% with a standard deviation of 5.3%.

Parity of the sow was divided in five classes: parity ‘1’ (N = 500), parity ‘2 to 3’ (N = 668), parity ‘4 to 5’ (N = 426), parity ‘6 to 7’ (N = 190) and parity ‘8+’ (N = 96).

Similarly, we estimated genetic correlations (r_g_) between traits using bivariate animal models as follows:

$$\left[\begin{array}{c}\mathbf{y}\boldsymbol{1}\\ \mathbf{y}\boldsymbol{2}\end{array}\right]=\left[\begin{array}{cc}\mathbf{X}\boldsymbol{1}& \boldsymbol{0}\\ \boldsymbol{0} & \mathbf{X}\boldsymbol{2}\end{array}\right]\left[\begin{array}{c}\mathbf{b}\boldsymbol{1}\\ \mathbf{b}\boldsymbol{2}\end{array}\right]+\left[\begin{array}{cc}\mathbf{Z}\boldsymbol{1}& \boldsymbol{0}\\ \boldsymbol{0}& \mathbf{Z}\boldsymbol{2}\end{array}\right]\left[\begin{array}{c}\mathbf{a}\boldsymbol{1}\\ \mathbf{a}\boldsymbol{2}\end{array}\right]+\left[\begin{array}{cc}\mathbf{W}\boldsymbol{1}& \boldsymbol{0}\\ \boldsymbol{0}& \mathbf{W}\boldsymbol{2}\end{array}\right]\left[\begin{array}{c}\mathbf{c}\boldsymbol{1}\\ \mathbf{c}\boldsymbol{2}\end{array}\right]+\left[\begin{array}{c}\mathbf{e}\boldsymbol{1}\\ \mathbf{e}\boldsymbol{2}\end{array}\right].$$

Similar to the single-trait animal model, $\mathbf{y}\boldsymbol{1}$ and $\mathbf{y}\boldsymbol{2}$ represent vectors with phenotypes for the studied traits; $\mathbf{b}\boldsymbol{1}$ and $\mathbf{b}\boldsymbol{2}$ are vectors of the fixed effects and covariates; $\mathbf{a}\boldsymbol{1}$ and $\mathbf{a}\boldsymbol{2}$ are vectors of additive genetic effects, assumed to follow a normal distribution for the $\mathbf{H}$ matrix using single-step genomic evaluation:

$$\left[\begin{array}{c}\mathbf{a}\boldsymbol{1}\\ \mathbf{a}\boldsymbol{2}\end{array}\right]\sim \text{N}\left(\left[\begin{array}{c}\boldsymbol{0}\\ \boldsymbol{0}\end{array}\right],\left[\begin{array}{cc}{\upsigma }_{\text{a}1}^{2}& {\upsigma }_{\text{a}1,\text{a}2}\\ {\upsigma }_{\text{a}1,\text{a}2}& {\upsigma }_{\text{a}2}^{2}\end{array}\right]\otimes \mathbf{H}\right).$$

$\mathbf{c}\boldsymbol{1}$ and $\mathbf{c}\boldsymbol{1}$ are vectors of common environmental pen effects, assumed to follow a normal distribution $$\left[\begin{array}{c}\mathbf{c}\boldsymbol{1}\\ \mathbf{c}\boldsymbol{2}\end{array}\right]\sim \text{N}(\left[\begin{array}{c}\boldsymbol{0}\\ \boldsymbol{0}\end{array}\right],\left[\begin{array}{cc}{\upsigma }_{\text{c}1}^{2}& {\upsigma }_{\text{c}1,\text{c}2}\\ {\upsigma }_{\text{c}1,\text{c}2}& {\upsigma }_{\text{c}2}^{2}\end{array}\right]\otimes \mathbf{I})$$; $\mathbf{e}\boldsymbol{1}$ and $\mathbf{e}\boldsymbol{2}$ are vectors of residual effects, assumed to follow a normal distribution $$\left[\begin{array}{c}\mathbf{e}\boldsymbol{1}\\ \mathbf{e}\boldsymbol{2}\end{array}\right]\sim \text{N}(\left[\begin{array}{c}\boldsymbol{0}\\ \boldsymbol{0}\end{array}\right],\left[\begin{array}{cc}{\upsigma }_{\text{e}1}^{2}& {\upsigma }_{\text{e}1,\text{e}2}\\ {\upsigma }_{\text{e}1,\text{e}2}& {\upsigma }_{\text{e}2}^{2}\end{array}\right])$$; $\mathbf{X}\boldsymbol{1}$, $\mathbf{X}\boldsymbol{2}$, $\mathbf{Z}\boldsymbol{1}$, $\mathbf{Z}\boldsymbol{2}$, $\mathbf{W}\boldsymbol{1}$ and $\mathbf{W}\boldsymbol{2}$ are incidence matrices for fixed effects, random animal effects and random common environmental pen effects, respectively.

## Results

### Association analysis of resilience traits with physical abnormalities and mortality

**Fig. 4:**
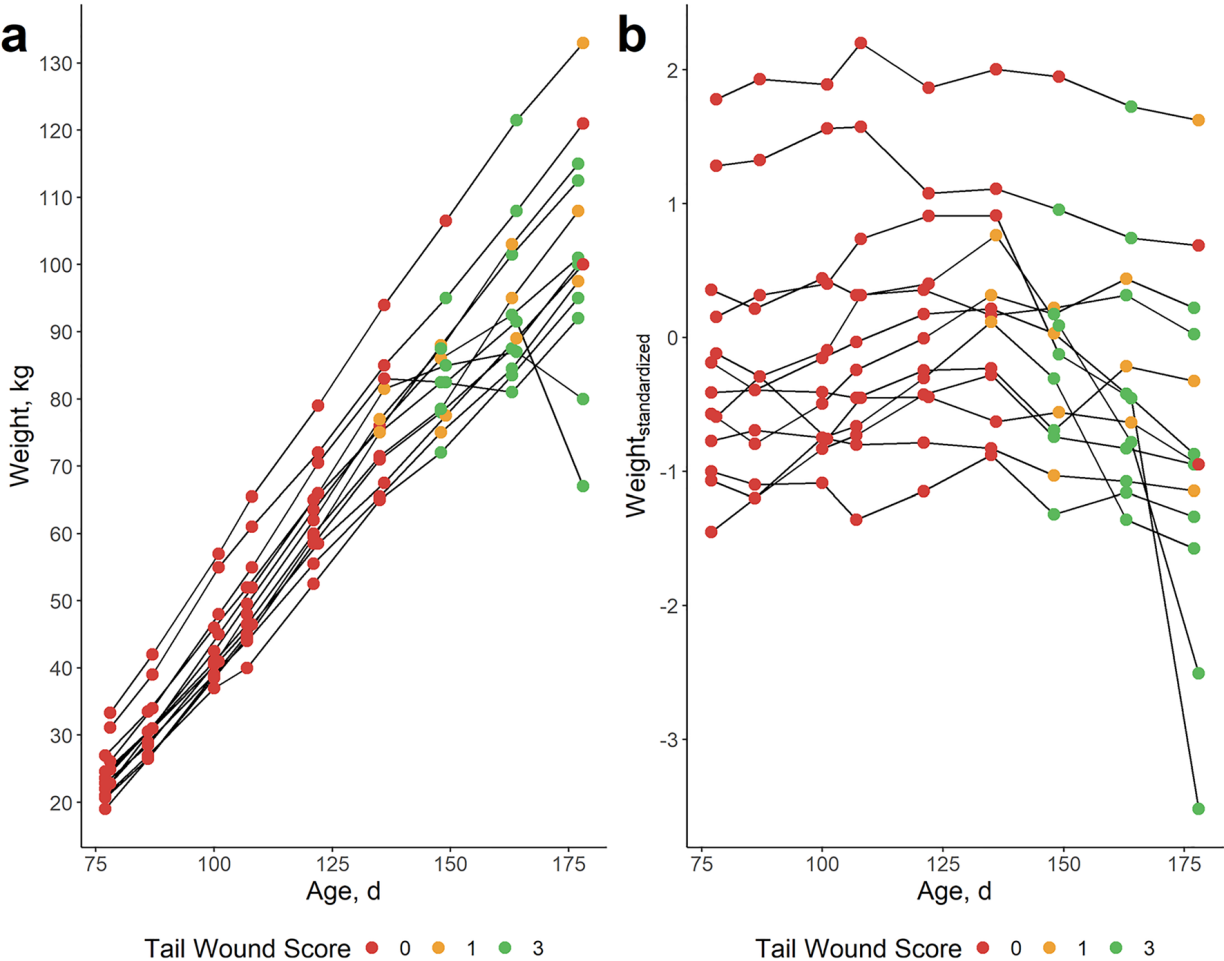
Example of weight evolution in one pen with an outbreak of tail biting around 140 days of age. Pigs with no tail lesions had a score 0 (red), those with small tail lesions had a score 1 (yellow) and those with severe tail lesions had a score 3 (green). **a** Weight evolution in kg and **b** evolution of standardized weights compared to population average per age bin of 3 days

The evolution of (standardized) weights according to age (in Fig. 1) shows that finishing pigs are more or less in their linear growth trajectory and most animals are within three standard deviations of the mean. The sizes of the effect of the association analysis of resilience traits according to mortality and physical abnormality scores are in Table 1. Based on these results, lnvar_weight_, lnvar_standardized_ and lnMSE_weight_ were strongly associated with mortality, lameness and tail biting wound scores. On average, pigs challenged by at least one of these conditions had significantly (p < 0.001) more deviations in body weight over time. This was also visible in the boxplots of resilience traits according to mortality and physical abnormality scores, which are shown in Fig. 3 for lnvar_weight_. For the other resilience traits, these boxplots are provided in Additional file 2 Figure S2, Additional file 3 Fig. 3, Additional file 4 Figure S4 and Additional file 5 Figure S5. These results show that tail biting wounds, lameness and mortality were associated with an increase in the deviations of predicted versus observed weight. An example of the association between tail biting wounds and lnvar_weight_ is shown in Fig. 4. In this specific pen, tail biting wounds started from an age of 140 days on all pigs, indicating an outbreak of tail biting. For two pigs, this led to a drastic decrease in body weight (Fig. 4a), while most pigs recovered after an initial decrease in body weight gain. At the pen level (Fig. 4b), standardized weights decreased after the tail biting event, meaning that the pigs’ weight relative to the population average decreased.

**Table 1:** Linear regression coefficients from the regression of physical abnormality traits on resilience traits

	Tail wound	Ear wound	Hematomas	Umbilical hernia	Lameness	Mortality
Lnvar_weight_	0.139***	0.063	0.001	− 0.202	0.818***	1.543***
Lnvar_standardized_	0.058**	0.104*	0.108°	− 0.038	0.680***	0.752***
lnMSE_weight_	0.110***	0.078°	0.024	− 0.047	0.854***	1.429***
lnMSE_order_	− 0.006	− 0.003	− 0.019	0.026	0.068	0.005
lnMSE_activity_	0.003	− 0.005	− 0.058	− 0.423*	0.040	0.344

Here, a score of ‘0’ is the reference, meaning an effect size of 0. For example, a score of ‘3’ for tail wounds implies an increase in lnMSE_weight_ of 3*0.110 or 0.330. Lnvar_weight_, lnvar_standardized_ and lnMSE_weight_ were all significantly associated with tail wounds, lameness and mortality. Boxplots of resilience trait scores according to physical abnormalities were drawn (Fig. 2) [see Additional file 2 Figure S2, Additional file 3 Figure S3, Additional file 4 Figure S4 and Additional file 5 Figure S5]. °p < 0.10; *p < 0.05; **p < 0.01; ***p < 0.001

Moreover, ear biting wounds were significantly associated with lnvar_standardized_ (p < 0.05), but not with lnvar_weight_ (p > 0.10) and lnMSE_weight_ (0.05 < p < 0.10). In contrast, deviations in weighing order (lnMSE_order_) and activity during weighing (lnMSE_activity_) were not associated with mortality and physical abnormalities (p > 0.10), except for a negative association between umbilical hernia and lnMSE_activity_ (p < 0.05).

The traits CV_start_, CV_middle_, CV_end_, FI and FCR were recorded at the pen level. Therefore, phenotypical correlations of these traits with individually recorded traits were estimated using the pen averages. The pairwise correlation matrix of the mean of the resilience traits at the pen level compared with traits recorded at the pen level is shown in Additional file 8 Figure S8. Most correlations were significantly different from zero and significance values of these correlations are also in Additional file 8 Figure S8. Interestingly, body weight deviations (lnvar_weight_, lnvar_standardized_, lnMSE_weight_) were phenotypically lowly correlated with CV at the pen level, especially at the end of the finishing phase (CV_end_) (r_p_ = 0.14 to 0.29). Moreover, lnvar_weight_, lnvar_standardized_ and lnMSE_weight_ had a negative correlation with FI (r_p_ = − 0.23 to − 0.14) and a positive correlation with FCR (r_p_ = 0.05 to 0.21), indicating that fewer deviations in body weight were lowly correlated with higher FI and lower FCR at the pen level. Correlations between body weight deviation traits and ADG_life_ were not consistent: lnvar_weight_ had a positive correlation (r_p_ = 0.10) with ADG_life_, whereas this correlation was negative after standardizing weights (r_p_ = − 0.10 between lnvar_standardized_ and ADG_life_) [Table 3 and Additional file 9 Figure S9].

### Genetic parameters

An overview of the estimated heritability and variance components is in Table 2. Gompertz curve parameters B and k had a moderate to high heritability (h^2^ = 40.3 and 47.3%). In contrast, the A parameter was estimated to be lowly heritable, but with a very high common environmental pen effect estimate (c^2^ = 91.2). Resilience traits from body weight deviations (lnvar_weight_, lnvar_standardized_ and lnMSE_weight_) were moderately heritable (h^2^ = 25.2 to 36.3%), but with very high GCV estimates of 44.5 to 59.5% (estimated from σ_a_). Moreover, when standardized weights were used the common environmental pen effect estimate was lower (c^2^ = 7.7% for lnvar_standardized_) compared to deviations in observed weights with Gompertz modeling (c^2^ = 8.4% for lnvar_weight_) and linear modeling (c^2^ = 14.3% for lnMSE_weight_). This indicates that scaling effects in observed weight over time might bias the estimates of weight deviations. Deviations in pigs’ weighing order (h^2^ = 4.2%) and activity during weighing over time (h^2^ = 12.0%) were lowly heritable and did not significantly differ from zero (using estimate ± 1.96*se). The heritability estimates for physical abnormality scores varied widely. Mortality (h^2^ = 6.1%), lameness (h^2^ = 8.3%) and umbilical hernia (h^2^ = 10.1%) had low heritability estimates. Tail wound score was moderately heritable (h^2^ = 16.4%), whereas hematomas (h^2^ = 41.6%) and ear wound scores (h^2^ = 46.3%) had high heritability estimates.

**Table 2:** Genetic parameter estimates

Trait	Mean (sd)	h^2^ (se)	c^2^ (se)	σ_a_	σ_c_	σ_e_
ADG_youth_ (kg/d)	0.319 (0.055)	60.0 (10.9)	20.7 (5.0)	0.044	0.026	0.025
ADG_life_ (kg/d)	0.625 (0.065)	68.4 (9.5)	9.1 (3.7)	0.057	0.021	0.033
A	180.7 (23.4)	4.5 (1.5)	91.6 (1.2)	5.281	23.757	4.872
B	5.75 (1.02)	40.3 (8.6)	37.2 (5.1)	0.606	0.582	0.452
k*1000	13.9 (2.8)	47.3 (9.5)	40.5 (5.4)	1.818	1.682	0.923
Meat percentage (%)	62.9 (3.0)	54.1 (10.0)	8.2 (3.5)	1.856	0.724	1.550
Lnvar_weight_	0.32 (0.97)	36.3 (8.3)	8.4 (3.3)	0.595	0.286	0.735
Lnvar_standardized_	− 2.20 (0.91)	30.6 (10.0)	7.7 (3.2)	0.513	0.258	0.729
lnMSE_weight_	0.77 (0.89)	25.2 (8.4)	14.3 (3.8)	0.445	0.335	0.688
lnMSE_activity_	3.31 (0.80)	12.3 (7.5)	2.4 (3.3)	0.277	0.124	0.740
lnMSE_order_	1.93 (0.60)	4.2 (4.8)	5.3 (2.3)	0.122	0.138	0.570
Tail wound score	1.04 (1.18)	16.4 (6.0)	53.5 (4.2)	0.478	0.865	0.649
Ear wound score	0.17 (0.49)	46.3 (9.7)	24.5 (5.0)	0.351	0.255	0.278
Hematomas score	0.10 (0.33)	41.6 (7.7)	0.2 (2.6)	0.221	0.017	0.261
Umbilical Hernia	0.02 (0.16)	10.1 (4.2)	1.2 (1.9)	0.050	0.017	0.148
Lameness	0.04 (0.19)	8.3 (4.1)	1.5 (1.8)	0.056	0.023	0.183
Mortality	0.03 (0.18)	6.1 (5.1)	38.1 (4.8)	0.049	0.123	0.149

Heritability (h^2^) and common environmental pen effect (c^2^) are given in percentages. Additive genetic standard deviation (σ_a_), common environmental standard deviation (σ_c_) and residuals standard deviation (σ_e_) are given in trait units. Estimates of the k-parameter of Gompertz modeling are multiplied by a factor 1000

An overview of the estimated phenotypic and genetic correlations is in Table 3. Genetic correlations of lnMSE_order_ and lnMSE_activity_ were not taken into account, as standard errors were very large. The resilience traits based on body deviations (lnvar_weight_, lnMSE_weight_ and lnvar_standardized_) were internally lowly to moderately correlated (r_p_ = 0.37 to 0.57; r_g_ = 0.11 to 0.66, se = 0.19 to 0.26). Interestingly, body weight deviations were genetically positively correlated with tail biting wounds (r_p_ = 0.09 to 0.21; r_g_ = 0.22 to 0.39, se = 0.25 to 0.36), lameness (r_p_ = 0.19 to 0.24; r_g_ = 0.15 to 0.44, se = 0.05 to 0.70) and mortality (r_p_ = 0.21 to 0.28; r_g_ = 0.19 to 0.43, se = 0.07 to 0.09). It should be noted that the estimated standard errors of these genetic correlations were quite high, with an average standard error of 0.24 and a range from 0.00 to 1.47 (see Additional file 10 Table S1). ADG_life_ was positively correlated with lnvar_weight_ (r_p_ = 0.10, r_g_ = 0.35, se = 0.18), but this positive correlation was not present after standardization of weights in lnvar_standardized_ (r_p_ = − 0.10, r_g_ = 0.02, se = 0.18). However, lnvar_standardized_ was positively correlated with meat percentage of the carcass (r_p_ = 0.09, r_g_ = 0.33, se = 0.18). Ear biting wound scores were positively correlated with tail biting wound scores (r_p_ = 0.19; r_g_ = 0.32, se = 0.33). Moreover, tail biting wound scores were also positively correlated with lameness (r_p_ = 0.12; r_g_ = 0.38, se = 1.07) and mortality (r_p_ = 0.12; r_g_ = 0.30, se = 0.12), and mortality and lameness were internally moderately to highly correlated (r_p_ = 0.32; r_g_ = 0.79, se = 0.13).

**Table 3:** Correlation table with estimated phenotypical (below the diagonal) and genetic correlations (above the diagonal)

Trait	ADG_youth_	ADG_life_	A	B	K	Meat percentage	Lnvar_weight_	Lnvar_standardized_	LnMSE_weight_	Tail_score_	Ear_score_	Hematomas	Hernia	Lameness	Mortality
ADG_youth_		0.72	− 0.48	0.16	0.39	− 0.18	0.32	0.05	− 0.15	− 0.05	0.02	− 0.04	0.05	0.18	0.02
ADG_life_	0.56		− 0.71	0.74	0.89	− 0.52	0.35	0.02	0.14	− 0.16	− 0.22	− 0.02	0.00	0.02	− 0.19
A	− 0.05	− 0.07		− 0.47	− 0.68	0.58	− 0.34	0.13	− 0.37	− 0.10	− 0.03	0.06	0.34	0.24	0.31
B	− 0.13	0.50	− 0.47		0.87	− 0.66	0.3	− 0.26	0.17	− 0.15	− 0.30	0.24	0.17	− 0.21	− 0.14
K	0.24	0.64	− 0.69	0.87		− 0.66	0.43	− 0.12	0.13	− 0.26	− 0.06	0.00	0.14	0.00	− 0.29
Meat percentage	− 0.13	− 0.52	0.12	− 0.48	− 0.48		− 0.07	0.33	− 0.22	0.02	0.04	− 0.02	0.05	0.16	–
Lnvar_weight_	0.18	0.10	− 0.06	− 0.06	0.04	− 0.03		0.32	0.66	0.39	− 0.02	− 0.22	− 0.02	0.44	0.43
Lnvar_standardized_	0.05	− 0.10	− 0.04	− 0.09	− 0.07	0.09	0.37		0.11	0.22	0.38	0.04	0.06	0.31	0.19
LnMSE_weight_	− 0.08	− 0.01	− 0.10	0.07	0.04	− 0.07	0.57	0.42		0.30	0.21	0.06	− 0.65	0.15	0.33
Tail_score_	0.02	− 0.04	0.10	− 0.14	− 0.14	− 0.05	0.21	0.09	0.21		0.32	− 0.17	− 0.06	0.38	0.30
Ear_score_	− 0.10	− 0.06	0.03	0.02	− 0.03	− 0.05	0.02	0.06	0.09	0.19		− 0.13	− 0.42	0.11	0.11
Hematomas	− 0.05	− 0.01	− 0.03	0.07	0.04	− 0.03	− 0.02	0.05	0.02	− 0.06	0.05		− 0.05	− 0.20	− 0.29
Hernia	− 0.06	− 0.03	− 0.03	0.02	− 0.01	− 0.02	− 0.01	0.02	0.02	− 0.06	− 0.03	− 0.01		− 0.20	− 0.29
Lameness	0.00	− 0.05	− 0.01	− 0.11	− 0.09	0.03	0.21	0.19	0.24	0.12	0.05	0.01	0.04		0.79
Mortality	− 0.03	–	0.02	− 0.13	− 0.11	–	0.27	0.21	0.28	0.12	0.02	0.00	0.08	0.32	

Standard errors of genetic correlations were also calculated with an average standard error of 0.24 and a range from 0.00 tot 1.47. [see Additional File 10, Table S1]. As pigs who died at the farm where not slaughtered, phenotypic correlations could not be estimated between mortality and ADG_life_, and between mortality and Meat% (indicated with “–”)

## Discussion

This study investigated the (genetic) relationship between resilience traits quantified from deviations in longitudinal data in pigs with traits related to pig welfare, health and resilience, such as lameness, tail biting wounds and mortality. These resilience traits are promising proxies for a general resilience trait in pigs, but currently evidence is scarce for this putative relationship. Our results showed a highly significant (p < 0.001) association and a positive genetic correlation between resilience traits derived from body weight deviations (lnvar_weight_, lnvar_standardized_ and lnMSE_weight_) and tail biting wounds, lameness and mortality, although standard errors of genetic correlations were high. Moreover, these resilience traits were moderately heritable and phenotypically lowly favourably correlated with FI and FCR at the pen level. Furthermore, individual body weight deviations were correlated with uniformity at the pen level just before slaughter (CV_end_), providing evidence that breeding for these resilience traits might increase pigs’ within-individual uniformity and resilience as well as within-family uniformity at the pen level. In summary, our results suggest that breeding for our proposed resilience traits based on body weight deviations would increase pigs’ general resilience, uniformity and FCR at the pen level. These findings are highly valuable for pig breeders, and might be transferable to other (livestock) species.

Heritability estimates of lnvar_weight_, lnvar_resilience_ and lnMSE_weight_ were moderate ranging from 25.2 to 36.3%. These estimates are on the high end compared to previous estimates for resilience parameters based on deviations in body weight (h^2^ = 3 to 31%) [22, 24, 25], deviations in feed intake (h^2^ = 7 to 31%) [20–23] and deviations in feeding behaviour (h^2^ = 16 to 40%) [21–23]. In addition, GCV estimates for these traits were very high in this study, ranging from 44.5 to 59.5%. In contrast, Homma et al. and Gorssen et al. [22, 23], respectively, reported GCV estimates ranging from 22 to 39% and 21 to 33% for resilience parameters based on deviations in body weight, feed intake and feeding behaviour. We argue that these high heritability estimates and genetic coefficient of variation estimates might be due to a combination of high standardization and a reasonably challenging environment in our study, as explained in a previous study [30]. All studied pigs were kept in the same pig building during 1 year, hence, environmental variation might have been relatively low across pens, possibly reducing common environmental and/or residual variance. Moreover, our finishing pigs were kept under commercial conditions, with a higher pig density and disease pressure compared to purebred pigs in high health breeding farms as was done in Revilla et al. [24].

A significant (p < 0.01) and positive relationship between resilience traits based on body weight deviations (lnvar_weight_, lnvar_resilience_ and lnMSE_weight_) and tail biting, lameness and mortality was found (Fig. 2 and Table 1) (Additional file 2 Figure S2, Additional file 3 Figure S3, Additional file 4 Figure S4, Additional file 5 Figure S5 and Additional file 6 Figure S6). Moreover, phenotypic and genetic correlations were also positive (r_p_ = 0.09 to 0.28; r_g_ = 0.15 to 0.44). These results indicate that breeding pigs for decreased deviations in body weight will also reduce tail biting, lameness and mortality. As such, these resilience traits might prove a valuable proxy for pig general health, welfare and resilience. Our positive correlation between deviations in body weight and mortality are consistent with Putz et al. [20] and Cheng et al. [26], who estimated moderate to high genetic correlations (r_g_ = 0.37 to 0.85) between deviations in daily feed intake and mortality and antibiotic treatments. To our knowledge, our study is the first to report a favourable association between resilience parameters derived from deviations in body weight and tail biting wounds and lameness. However, several studies previously mentioned that average daily weight gain can be temporarily seriously affected in tail-bitten pigs [45–48] and in pigs suffering lameness [49].

We have to note that in our study, the CV_start_, CV_middle_, CV_end_, FI and FCR traits were recorded at the pen level. Therefore, phenotypical correlations of these traits with individually recorded traits were estimated using the pen averages. Using this methodology, we found a positive correlation between resilience indicators from body weight deviations and coefficient of variation at the end of the finishing phase (r_p_ = 0.14 to 0.29), showing that deviations on the individual level are also lowly to moderately correlated with uniformity at the pen level. These findings demonstrate that breeding pigs for an increased resilience based on individual body weight deviations (i.e., within-individual uniformity) could also improve the uniformity of pigs’ weight at the pen level (i.e., within-family uniformity). However, we were not able to estimate genetic correlations between within-individual uniformity and within-family uniformity due to the limited number of pens (N = 151). Future research could investigate this putative genetic relationship more in detail. In addition, lnvar_weight_, lnvar_standardized_ and lnMSE_weight_ were also favourably correlated with FI (r_p_ = − 0.23 to − 0.14) and FCR (r_p_ = 0.05 to 0.21), implying that an increase in body weight deviations (lower resilience) is associated with a decreased FI and increased FCR at the pen level. Gorssen et al. [22] reported a similar favorable relationship between FCR and lnvar_weight_ (r_p_ = 0.17 and r_g_ = 0.37) at the individual level, although they reported an unfavorable genetic correlation between FI and lnvar_weight_ (r_p_ = − 0.03 and r_g_ = 0.26). As FI and FCR were recorded at the pen level, genetic correlations were not estimated due to the limited amount of pens (N = 151).

In contrast to deviations in body weight, lnMSE_order_ and lnMSE_activity_ had low heritability estimates (h^2^ = 4.2% and 12.0%), which did not significantly differ from zero. No clear association was found between lnMSE_activity_ and resilience-related traits, such as mortality. This is somewhat in contrast with the results of van der Zande et al. [27], who found a phenotypical association between deviations in activity levels and disease status and morbidity in pigs three days after infection with the porcine reproductive and respiratory syndrome virus (PRRSV).

Heritability estimates for tail wound scores (h^2^ = 16.4%) were low to moderate, and comparable to other studies reporting heritabilities for tail lesions, such as Martinsen et al. (h^2^ = 5%) [50], Hermesch et al. (h^2^ = 9 to 25%) [51] and a study on clinical tail biters by Breuer et al. (h^2^ = 0 to 27%) [52]. In our study, tail wound scores were also positively correlated with ear biting wound scores (r_p_ = 0.19; r_g_ = 0.32), which intuitively makes sense and was also found in previous studies [53–56]. Furthermore, ear biting wound scores and hematomas were estimated to be moderately to highly heritable (h^2^ = 46.3 and 41.6%). These results are interesting, as little is known about the heritability of these traits in pigs. Estimating heritabilities for ear and tail biting is challenging, as it is difficult to distinguish victims from biters and neutral pigs. In our study, however, every pen (~ 13 pigs) consisted of a mixture of half-sibs and full-sibs that originated from the same Piétrain sire and one to three crossbred dams. Therefore, pens with an incidence of tail and/or ear biting wounds had a similar genetic background, making it possible to estimate genetic variances for the occurence of biting behaviour, in general. However, this experimental design also complicated genetic modeling and can affect the accuracy of the estimates. We were quite rigid in our statistical modelling and used a random common environmental pen effect, although this pen-effect might partly overlap with the genetic component. Nevertheless, heritability estimates were significantly higher than zero. Litter effects were not included in the genetic model, as we believe this would overlap with genetic effects. A model including a random effect of the litter seemed to overestimate heritabilities for tail biting wounds (h^2^ = 77.3%) and ear biting wounds (h^2^ = 89.9%), whereas a model with both litter and pen as random effects experienced convergence issues (detailed results not shown). However, as we used an animal model and ssGBLUP while correcting for common pen effects, litter effects will be at least partially captured via the **H**-matrix. Within pens, the average genetic relatedness estimated from the **H**-matrix across individuals was 42.9%, with a mean standard deviation of 10.8%. Moreover, within the studied set of 1919 finishing pigs the average genetic relatedness was 7.6% with a standard deviation of 5.3%. Therefore, we argue that there was sufficient variability in relationships among pigs to accurately estimate genetic parameters.

No clear association was found with our resilience traits and ear biting wounds, although lnvar_standardized_ was significantly associated with ear biting wounds (p < 0.05) and had a positive genetic correlation (r_p_ = 0.06; r_g_ = 0.38). In our study, we observed that pigs usually already showed ear biting lesions upon arrival in the finishing farm. Only a few pigs showed new ear biting wounds after arrival, suggesting that this behaviour originated mostly in the farrowing and/or nursery farm. Although no data were available on the precise age of onset of ear biting in this study, previous studies [56] reported that ear biting behaviour often starts during the weaner stage (5–12 weeks of age) and mostly within a few weeks post-weaning. We observed that the prevalence of tail biting wounds increased at an age of approximately 140–160 days in our study. This might also explain why ear biting wounds do not show a relationship with all deviation in body weight traits in our dataset: as the ear biting challenge was mostly initiated before weight recording, the weight deviations might have occured before arrival at the finishing farm. The same reasoning might be applicable to ear hematomas: most hematomas originated early in life. This is in line with [57], who reported the highest incidence of hematomas 4 to 5 weeks after weaning. Therefore, we hypothesise that ear biting wounds and ear hematomas in pigs might be related with deviations in early-life body weight. For example, [57, 58] indicated that ear hematomas can temporarily negatively impact average daily gain up to 2 weeks after incidence, followed by compensatory growth. However, future research is necessary to test this hypothesis. This hypothesis also implies that our proposed resilience traits might be sensitive to the observation period, as indicated in Berghof et al. [2] and shown in Gorssen et al. [22]. Hence, deviations in body weight during the finishing phase might genetically differ from deviations in body weight during early-life in pigs. Furthermore, no significant association was found between our resilience parameters and umbilical hernias. Possibly, this is because umbilical hernias are a partly heritable congenital effect with a gradual development over a pigs’ lifetime. As a result, the presented challenge of an umbilical hernia might lower the average weight gain of pigs as observed in Searcy-Bernal et al. [59] and Straw et al. [60], but it might not cause substantial short-term deviations in observed versus expected weight. In addition, piglets with a umbilical hernia are generally excluded for progeny testing, and this preselection effect might also have influenced this relationship.

Although our proposed resilience traits lnvar_weight_ and lnMSE_weight_ are promising, it is important to note that these traits are based on deviations of observed versus expected weights using a model (Gompertz versus linear modeling). As indicated in a previous paper by Gorssen et al. [22], modeling theoretical weights based on observations is difficult, certainly for severely challenged animals (as can be seen in Fig. 2d, for example). Therefore, lnvar_standardized_ may be preferred as a resilience trait, as this trait does not rely upon a theoretical model, but reflects longitudinal changes of an individual animal’s weight compared to the population average. Moreover, Gorssen et al. [22] showed that lnvar_standardized_ was more robust to a low data density and standardizes the natural increase of body weight variance over time, as can be seen in Fig. 1a and b.

The proposed resilience traits have the advantage over recording physical abnormality traits only that they offer a way to refine phenotypes and allow a holistic approach [4]. Many resilience-related traits are recorded as binary (e.g. ‘lame’ vs not ‘lame’) or ordinal variables (e.g. tail wound score), although the underlying biological mechanisms are most probably complex and continuous [61]. As Fig. 3 shows, much of the variation in lnvar_weight_ is still present in pigs without any visible physical abnormality. Pigs with a high lnvar_weight_ score, but without visible deficiencies might be presented with other challenges, such as infections and/or disease, thermal discomfort, social stressors and/or bullying. However, we did not record these traits in the present study. Challenged pigs also showed great variation in lnvar_weight_ scores. Some pigs with severe tail biting wounds, for example (Figs. 3 and 4), did not show much body weight deviations, indicating they might be more resilient to this stressor, whereas others were severely affected. Future studies should focus on this complex relationship.

For pig breeders, manually obtaining repeated records of pigs’ body weight records as was done in this study might be too costly and practically infeasible. However, new precision livestock technologies are developed with unprecedented speed, facilitating the collection of longitudinal data. For example, automated feeding stations are already used by most pig breeding companies. Furthermore, computer vision and wearable devices would allow to monitor almost continuously phenotypes such as body composition [17], tail biting events [18], heart rate, respiration rate and body temperature [19]. As a result, our findings will be valuable for pig breeders, as they show that resilience indicators based on deviations in longitudinal body weight in finishing pigs are heritable and associated with pig general health, welfare and resilience.

## Conclusions

This study showed that resilience traits derived from deviations in body weight are promising proxies for a pig’s general health, welfare and resilience. Our results showed a highly significant (p < 0.001) association between body weight deviation traits lnvar_weight_, lnvar_standardized_ and lnMSE_weight_ and tail biting wounds, lameness and mortality. Moreover, these resilience traits were moderately heritable and also genetically correlated with tail biting wounds, lameness and mortality. Furthermore, individual body weight deviations were positively correlated with uniformity at the pen level, providing evidence that breeding for these resilience traits might also increase pigs’ within-family uniformity. Our findings will be valuable for pig breeders, as they provide evidence that resilience indicators from deviations in body weight in finishing pigs are heritable and associated with pig general health, welfare and resilience**.**

### Supplementary Information

**Additional file 1: Figure S1.** Scoring sheet for physical abnormalities. Graphic representation of the scoring method of physical abnormalities in finishing pigs during the experiment. The scored abnormalities were tail biting wounds, ear biting wounds, ear hematomas or ear swellings and umbilical hernia.

**Additional file 2: Figure S2.** Boxplots of lnvar_standardized_ according to mortality and physical abnormality scores. Boxplots of lnvar_standardized_ according to mortality and physical abnormality scores. Small dots indicate scores for individual pigs. Effect sizes and significance of these differences were statistically tested and are in Table 1.

**Additional file 3: Figure S3.** Boxplots of lnMSE_weight_ according to mortality and physical abnormality scores. Boxplots of lnMSE_weight_ according to mortality and physical abnormality scores. Small dots indicate scores for individual pigs. Effect sizes and significance of these differences were statistically tested and are in Table 1.

**Additional file 4: Figure S4.** Boxplots of lnMSE_order_ according to mortality and physical abnormality scores. Boxplots of lnMSE_order_ according to mortality and physical abnormality scores. Small dots indicate scores for individual pigs. Effect sizes and significance of these differences were statistically tested and are in Table 1.

**Additional file 5: Figure S5.** Boxplots of lnMSE_activity_ according to mortality and physical abnormality scores. Boxplots of lnMSE_activity_ according to mortality and physical abnormality scores. Small dots indicate scores for individual pigs. Effect sizes and significance of these differences were statistically tested and are in Table 1.

**Additional file 6: Figure S6.** Pairwise correlation plots of resilience traits according to physical abnormality scores at the individual level. Pairwise correlation plots for all evaluated resilience traits according to mortality and physical abnormality scores, based on individual scores. Below the diagonal the pairwise correlation plots are shown. Above the diagonal Pearson correlations are shown. °: correlation is significantly different from zero with p < 0.10. *: correlation is significantly different from zero with p < 0.05. **: correlation is significantly different from zero with p < 0.01. ***: correlation is significantly different from zero with p < 0.001.

**Additional file 7: Figure S7.** Pairwise correlation plots of resilience traits according to physical abnormality scores at the pen level. Pairwise correlation plots for all evaluated resilience traits according to mortality and physical abnormality scores, based on the mean score at the pen level. Below the diagonal the pairwise correlation plots are shown. Above the diagonal Pearson correlations are shown. °: correlation is significantly different from zero with p < 0.10. *: correlation is significantly different from zero with p < 0.05. **: correlation is significantly different from zero with p < 0.01. ***: correlation is significantly different from zero with p < 0.001.

**Additional file 8: Figure S8.** Pairwise correlation plots of resilience traits according to uniformity, feed intake and feed conversion ratio at the pen level. Pairwise correlation plots for all evaluated resilience traits according to coefficient of variation (CV) at the start, middle and end of the finishing period, as well as the feed conversion ratio. As these traits were recorded at the pen level, the pen-mean of the resilience trait was calculated. Below the diagonal the pairwise correlation plots are shown. Above the diagonal Pearson correlations are shown. °: correlation is significantly different from zero with p < 0.10. *: correlation is significantly different from zero with p < 0.05. **: correlation is significantly different from zero with p < 0.01. ***: correlation is significantly different from zero with p < 0.001.

**Additional file 9: Figure S9.** Pairwise correlation plots of resilience traits according to production traits at the individual level. Pairwise correlation plots within all evaluated resilience traits and between resilience traits and production traits, such as the Gompertz growth curve parameters (A, B, k), average daily gain at start (ADG_youth_) and over the pigs life (ADG_life_), the recorded meat percentage at slaughterhouse, mean feed intake at pen level (FI) and mean feed conversion ratio at the pen level (FCR). Note that the correlations with FI and FCR might be inflated here, as these were recorded at the pen level, but correlated with traits recorded at the individual level. The correlations at the pen level, without possible inflation of the correlation were also constructed (see Additional file 8 Figure S8). Below the diagonal the pairwise correlation plots are shown. Above the diagonal Pearson correlations are shown. °: correlation is significantly different from zero with p < 0.10. *: correlation is significantly different from zero with p < 0.05. **: correlation is significantly different from zero with p < 0.01. ***: correlation is significantly different from zero with p < 0.001.

**Additional file 10: Table S1.** Genetic correlations between all trait combinations using bivariate models. Genetic correlations between all trait combinations using bivariate models. Columns A (Trait1) and B (Trait2) give the trait names used in the bivariate animal model. Columns C (h2_trait1) and D (h2_trait2) show the estimated heritability for trait 1 and trait 2. Column E (genetic_correlation) shows the estimated genetic correlation, with the estimated standard error in column F (SE_genetic_correlation). Genetic correlations of lnMSE_order_ and lnMSE_activity_ were not taken into account, as standard errors were very high.

## Data Availability

The datasets generated and/or analysed during the current study are not publicly available due to data restriction from the Vlaamse Piétrain Fokkerij vzw but are available from the corresponding author on reasonable request and with permission of the Vlaamse Piétrain Fokkerij vzw.
