# Supplementary figures and images for: Breeding for resilience in finishing pigs can decrease tail biting, lameness and mortality
Source: Genet Sel Evol. 2024 Jun 20;56:48. doi: 10.1186/s12711-024-00919-1 (PMC11191330; doi:10.1186/s12711-024-00919-1)

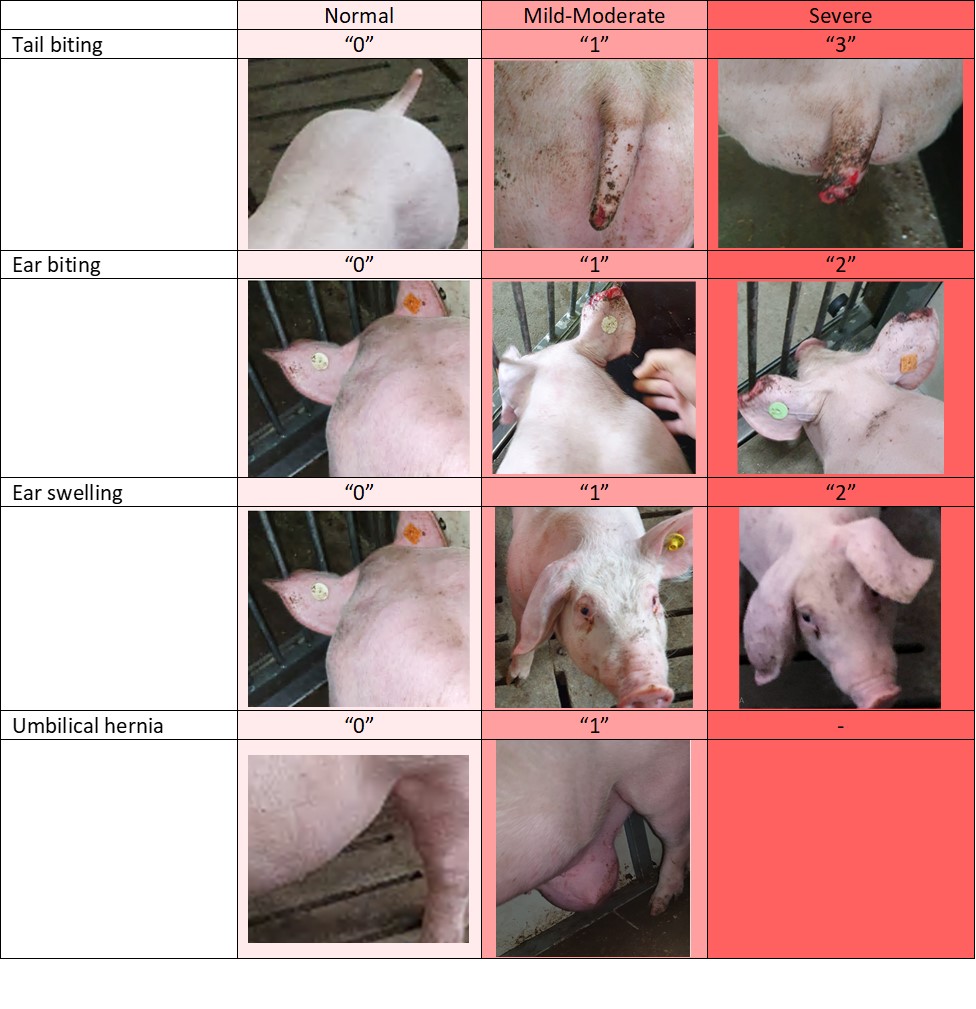

Supplement: Supplementary file 1 — Additional file 1: Figure S1. Scoring sheet for physical abnormalities. Graphic representation of the scoring method of physical abnormalities in finishing pigs during the experiment. The scored abnormalities were tail biting wounds, ear biting wounds, ear hematomas or ear swellings and umbilical hernia. [file 12711_2024_919_MOESM1_ESM.jpg]

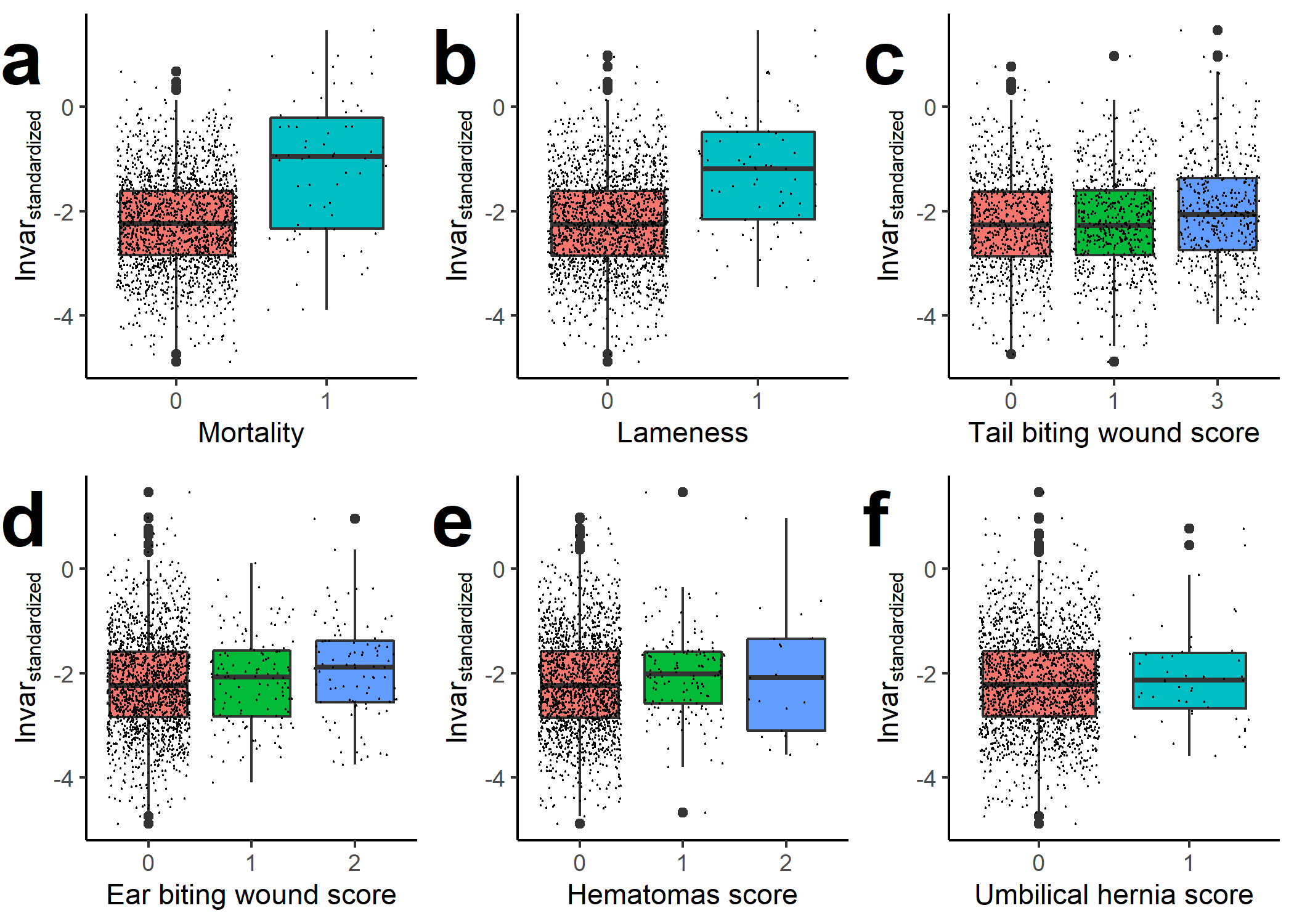

Supplement: Supplementary file 2 — Additional file 2: Figure S2. Boxplots of lnvarstandardized according to mortality and physical abnormality scores. Boxplots of lnvarstandardized according to mortality and physical abnormality scores. Small dots indicate scores for individual pigs. Effect sizes and significance of these differences were statistically tested and are in Table 1. [file 12711_2024_919_MOESM2_ESM.png]

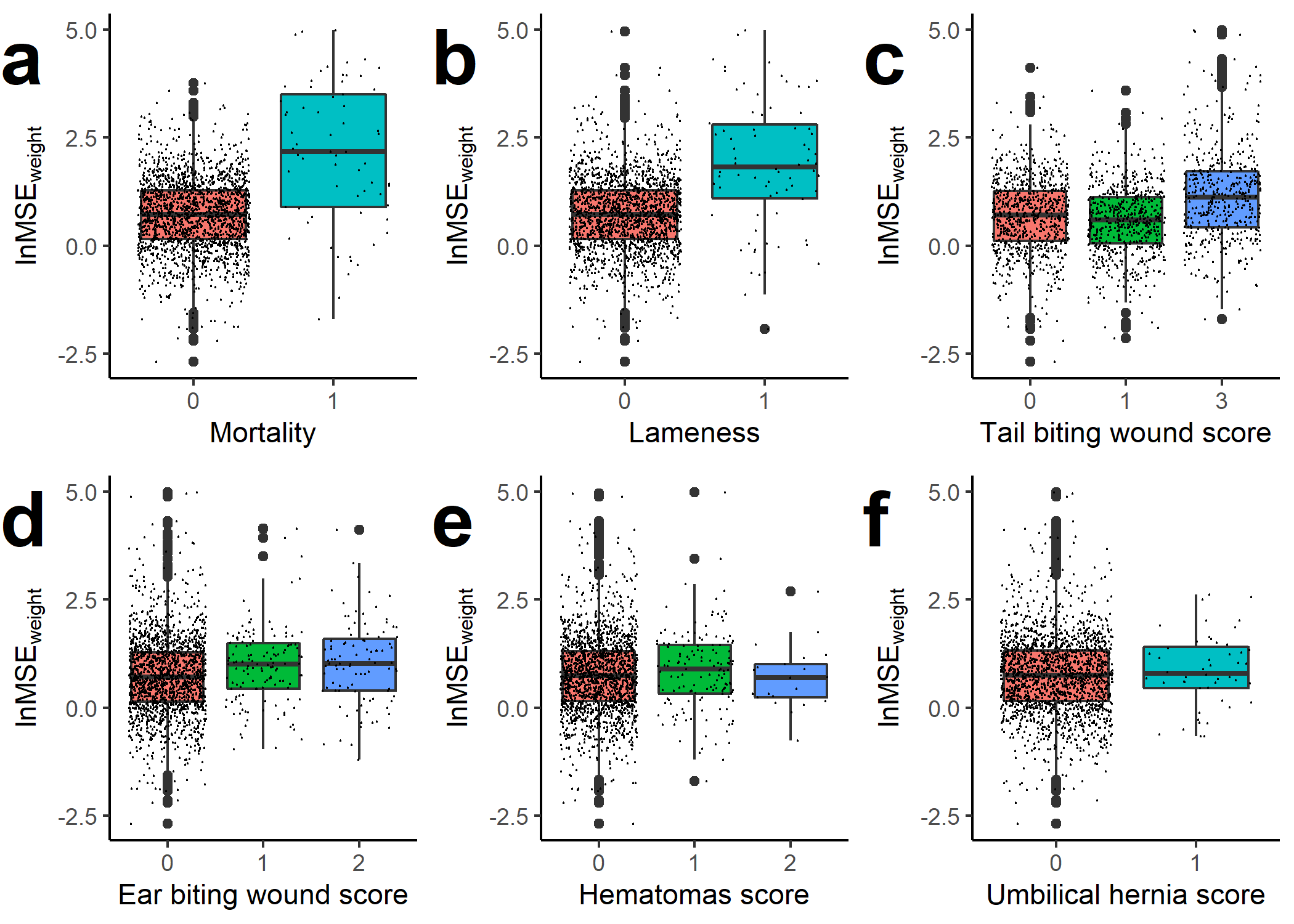

Supplement: Supplementary file 3 — Additional file 3: Figure S3. Boxplots of lnMSEweight according to mortality and physical abnormality scores. Boxplots of lnMSEweight according to mortality and physical abnormality scores. Small dots indicate scores for individual pigs. Effect sizes and significance of these differences were statistically tested and are in Table 1. [file 12711_2024_919_MOESM3_ESM.png]

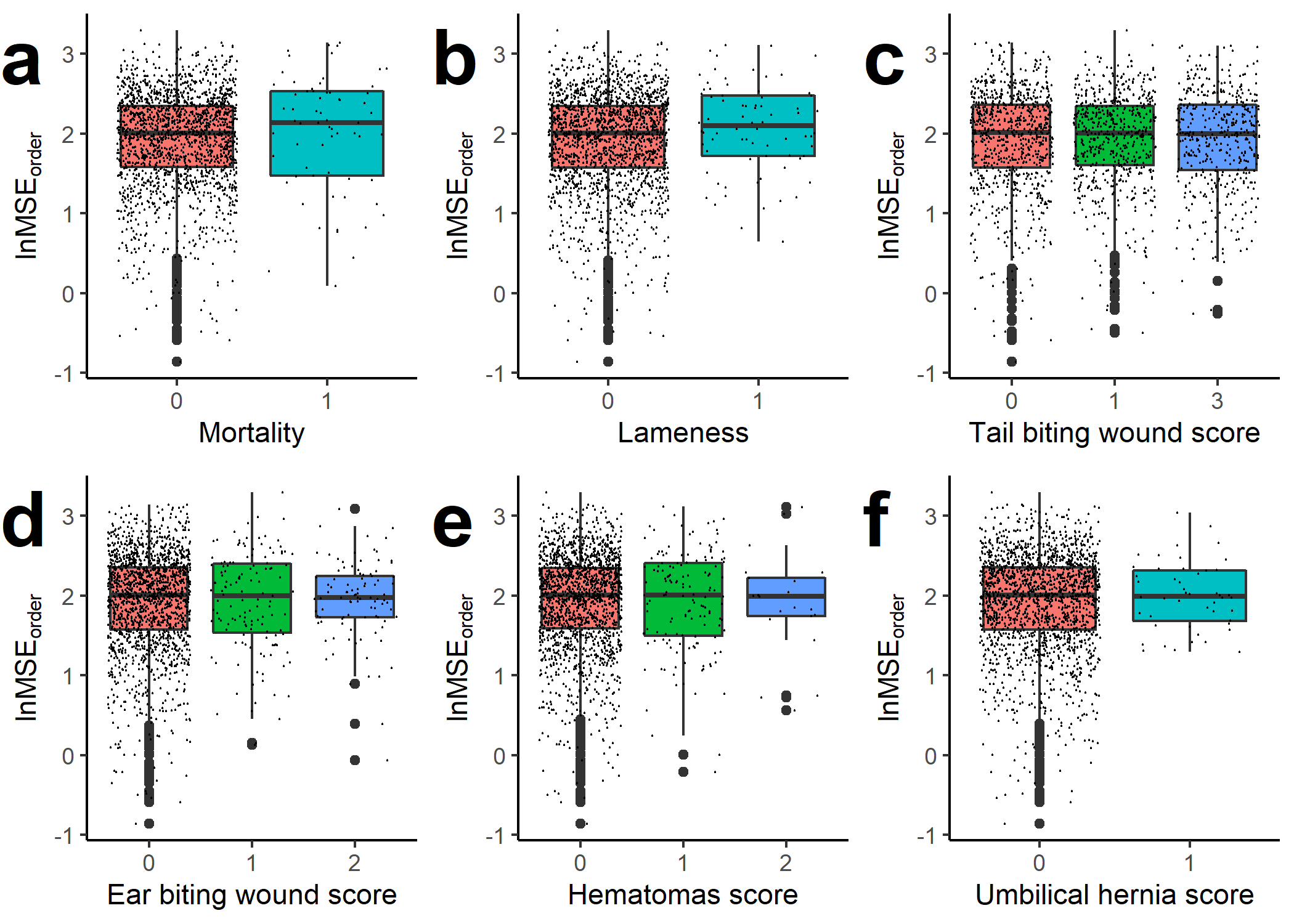

Supplement: Supplementary file 4 — Additional file 4: Figure S4. Boxplots of lnMSEorder according to mortality and physical abnormality scores. Boxplots of lnMSEorder according to mortality and physical abnormality scores. Small dots indicate scores for individual pigs. Effect sizes and significance of these differences were statistically tested and are in Table 1. [file 12711_2024_919_MOESM4_ESM.png]

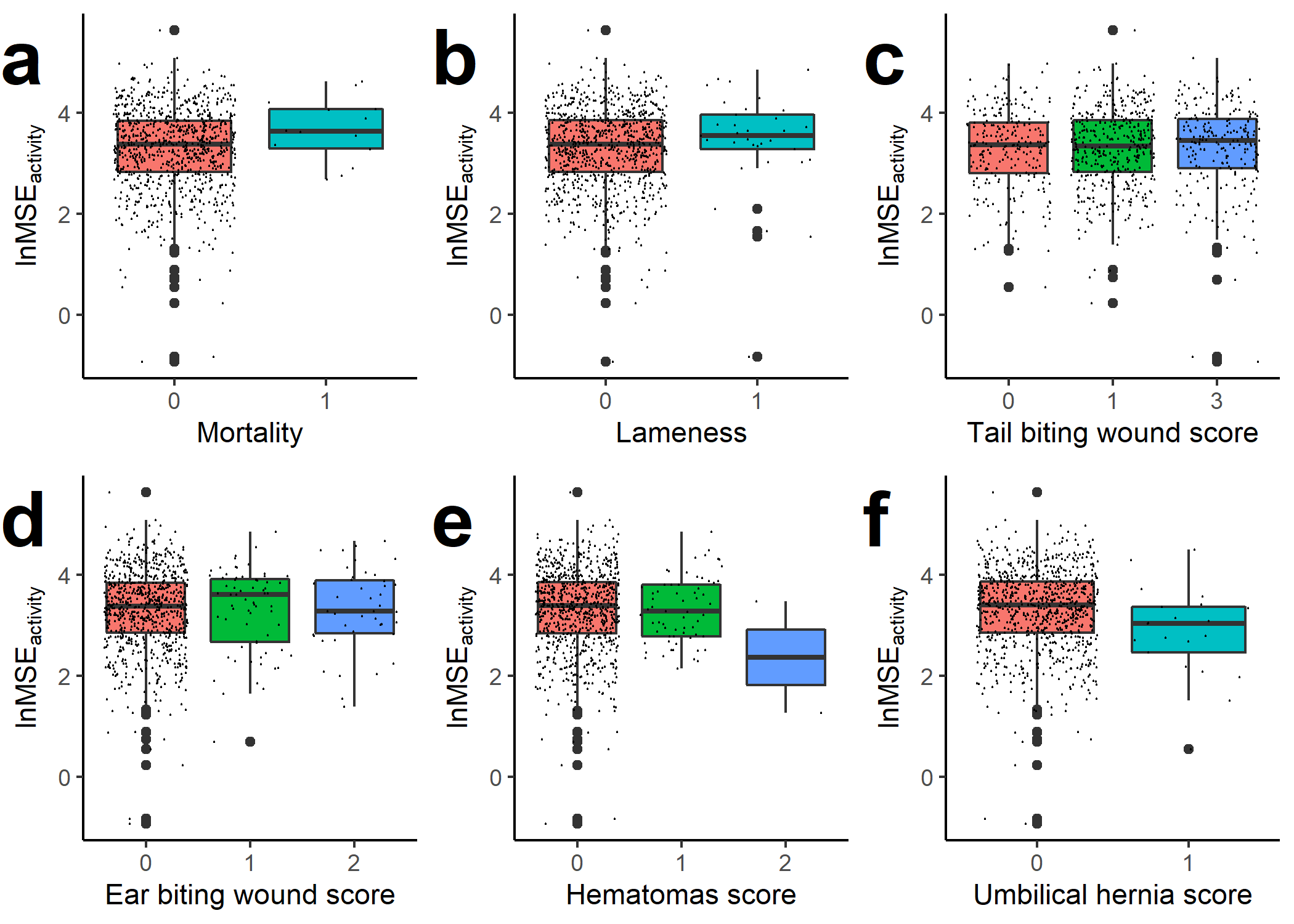

Supplement: Supplementary file 5 — Additional file 5: Figure S5. Boxplots of lnMSEactivity according to mortality and physical abnormality scores. Boxplots of lnMSEactivity according to mortality and physical abnormality scores. Small dots indicate scores for individual pigs. Effect sizes and significance of these differences were statistically tested and are in Table 1. [file 12711_2024_919_MOESM5_ESM.png]

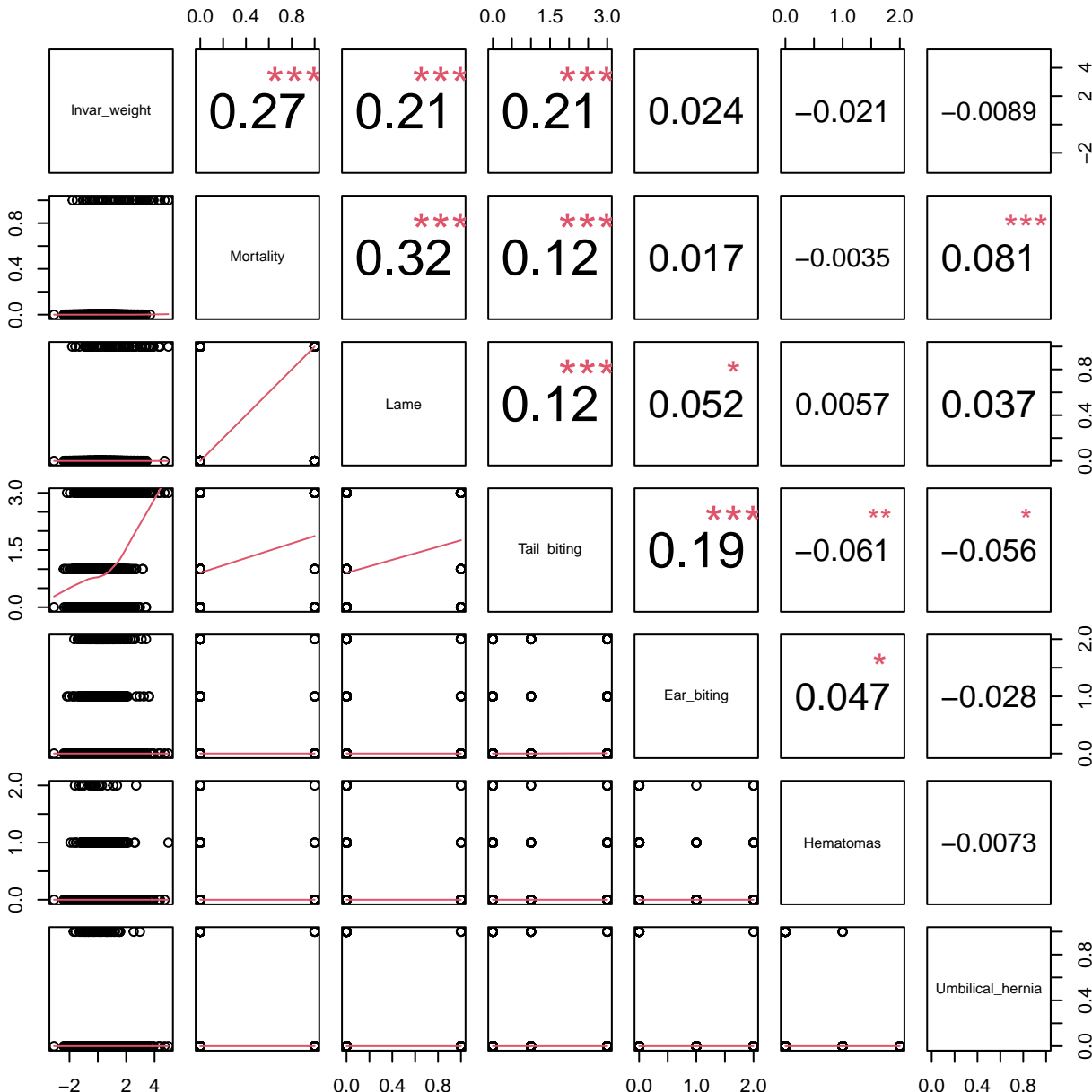

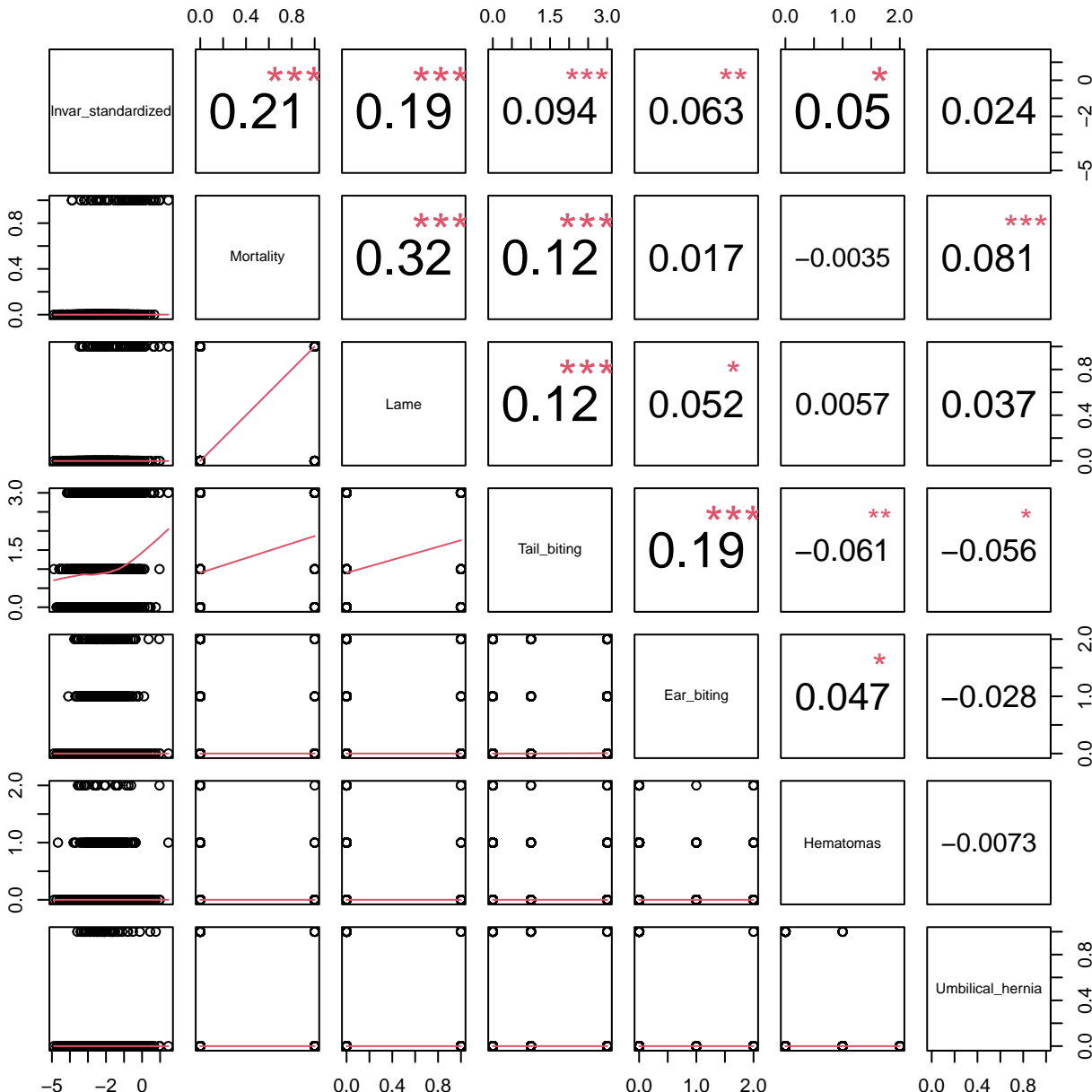

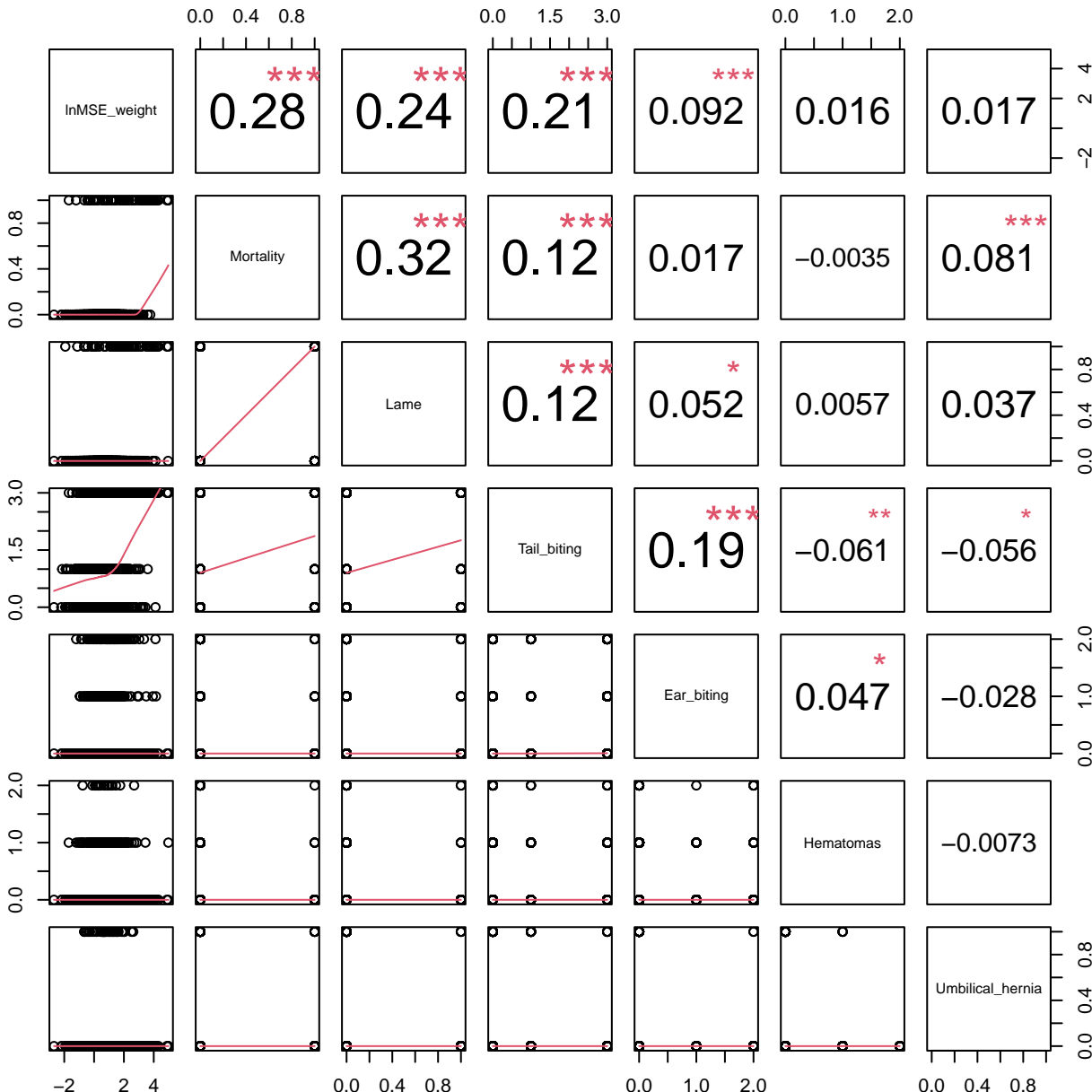

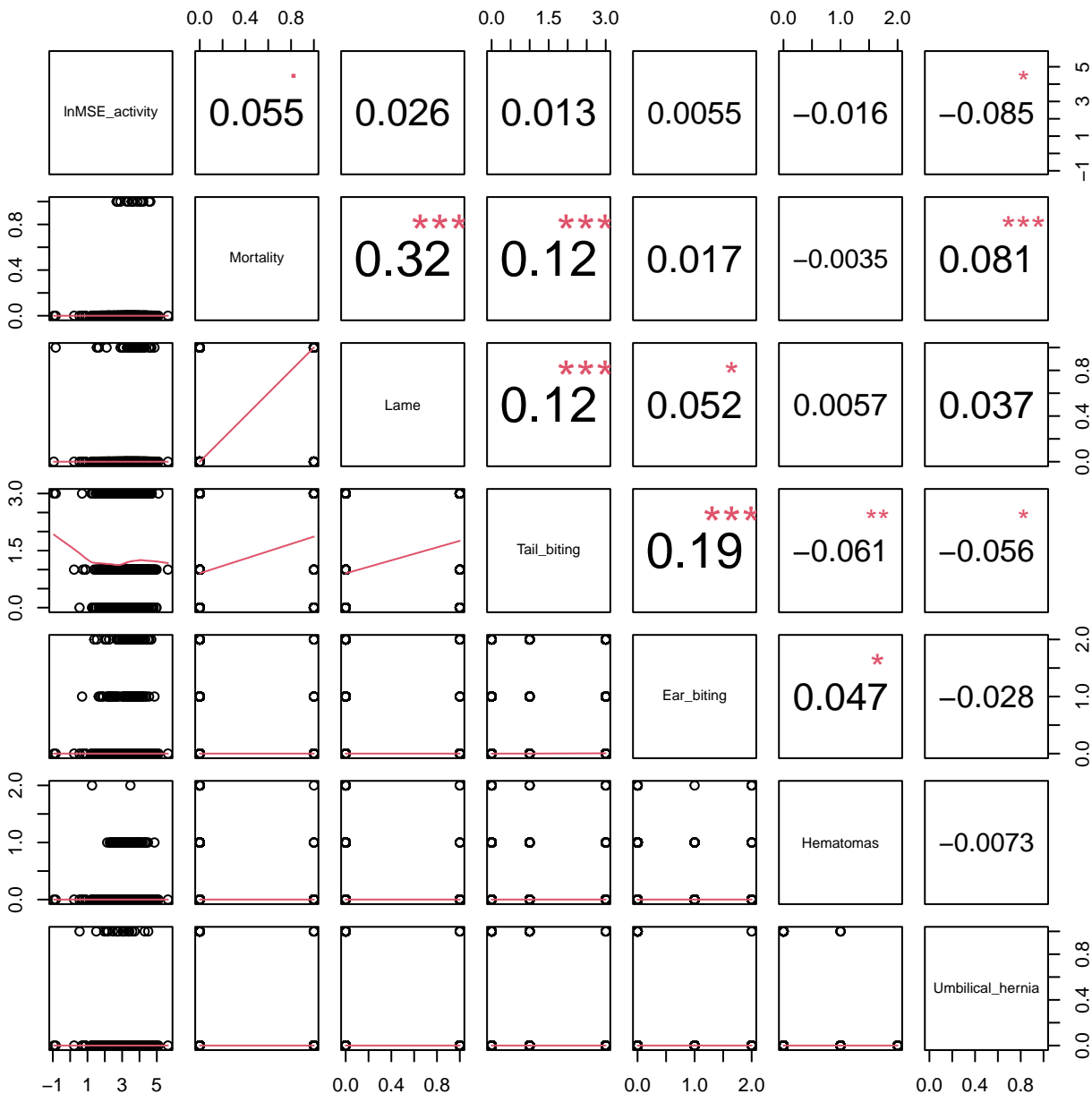

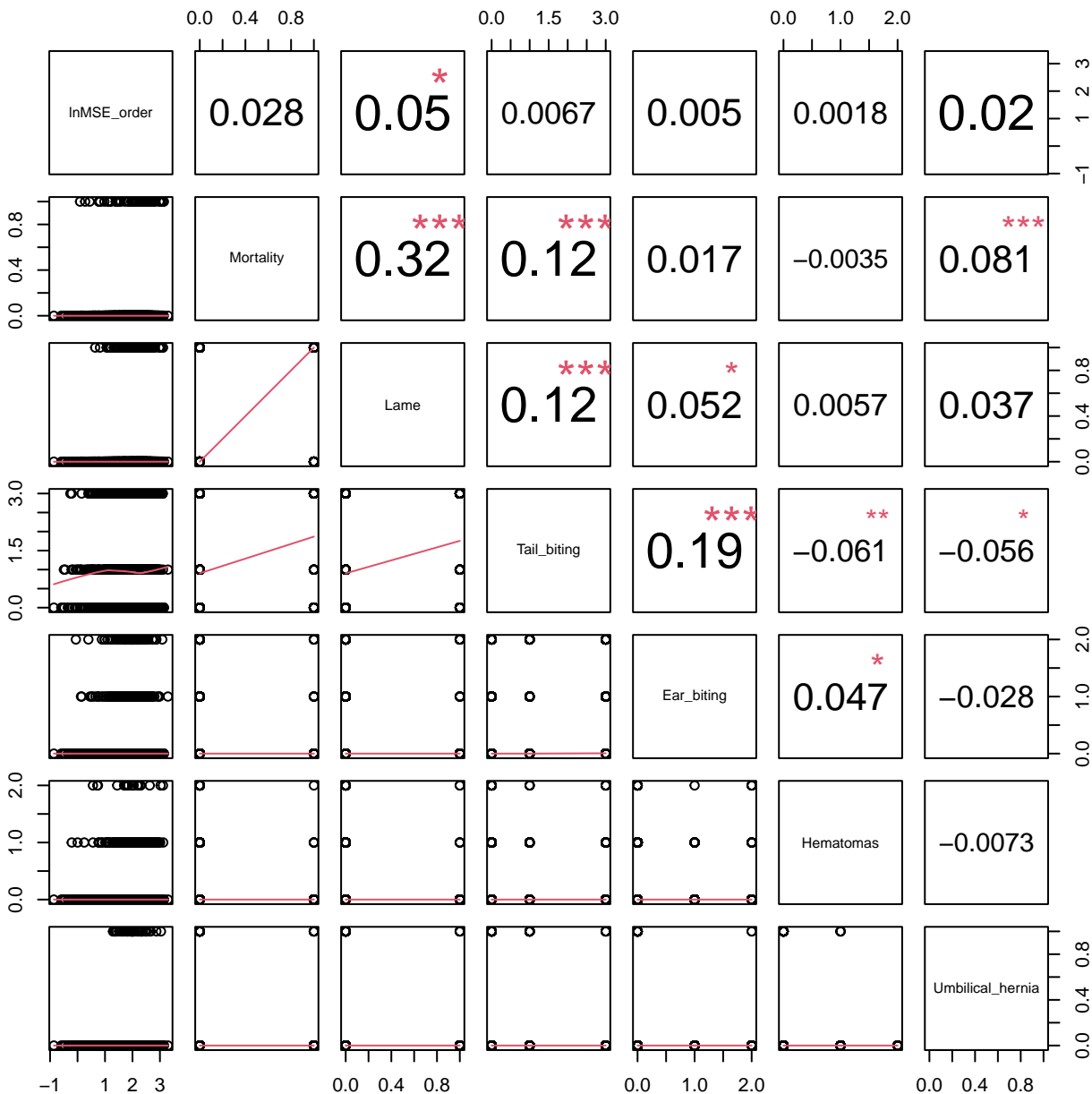

Supplement: Supplementary file 6 — Additional file 6: Figure S6. Pairwise correlation plots of resilience traits according to physical abnormality scores at the individual level. Pairwise correlation plots for all evaluated resilience traits according to mortality and physical abnormality scores, based on individual scores. Below the diagonal the pairwise correlation plots are shown. Above the diagonal Pearson correlations are shown. °: correlation is significantly different from zero with p < 0.10. *: correlation is significantly different from zero with p < 0.05. **: correlation is significantly different from zero with p < 0.01. ***: correlation is significantly different from zero with p < 0.001. [file 12711_2024_919_MOESM6_ESM.pdf]

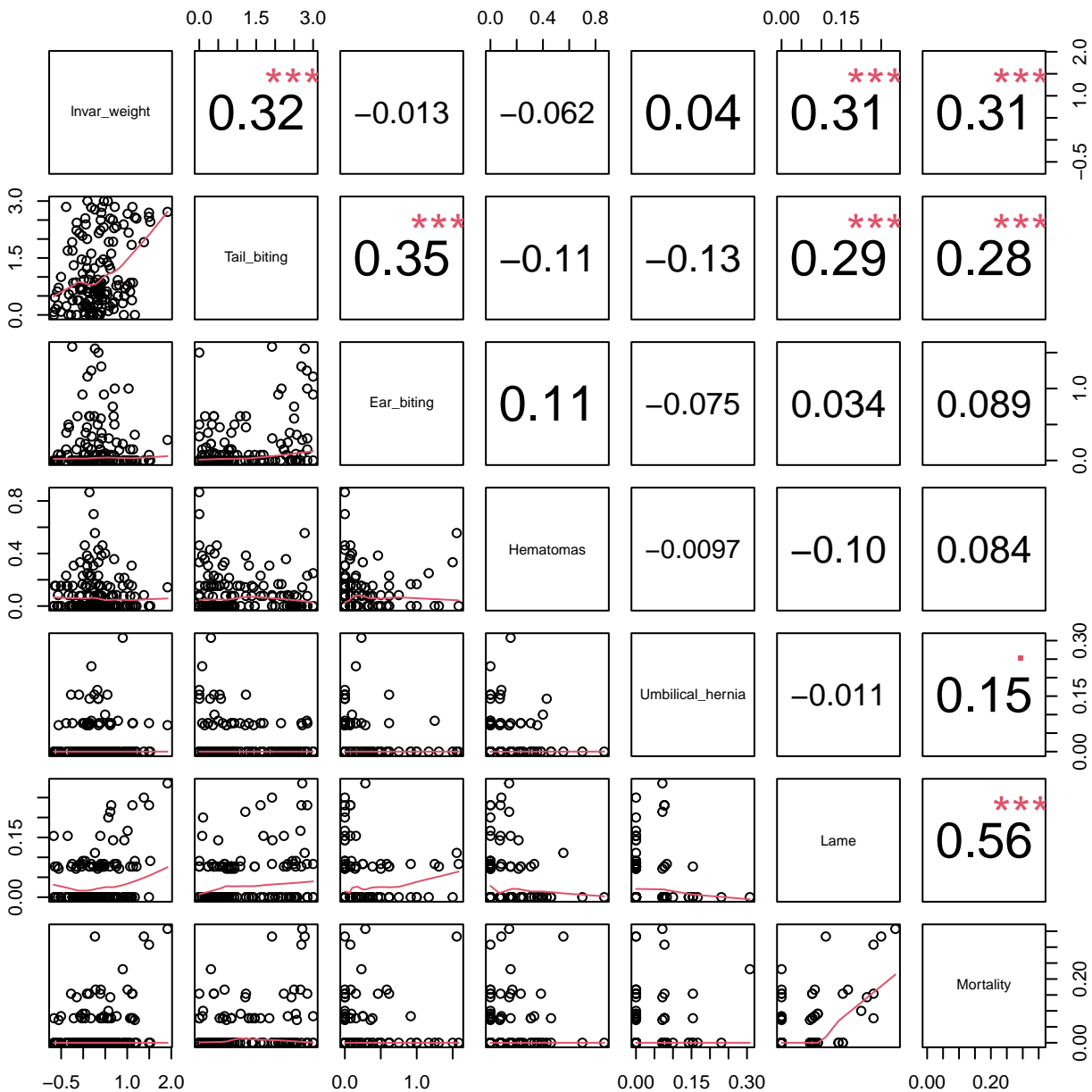

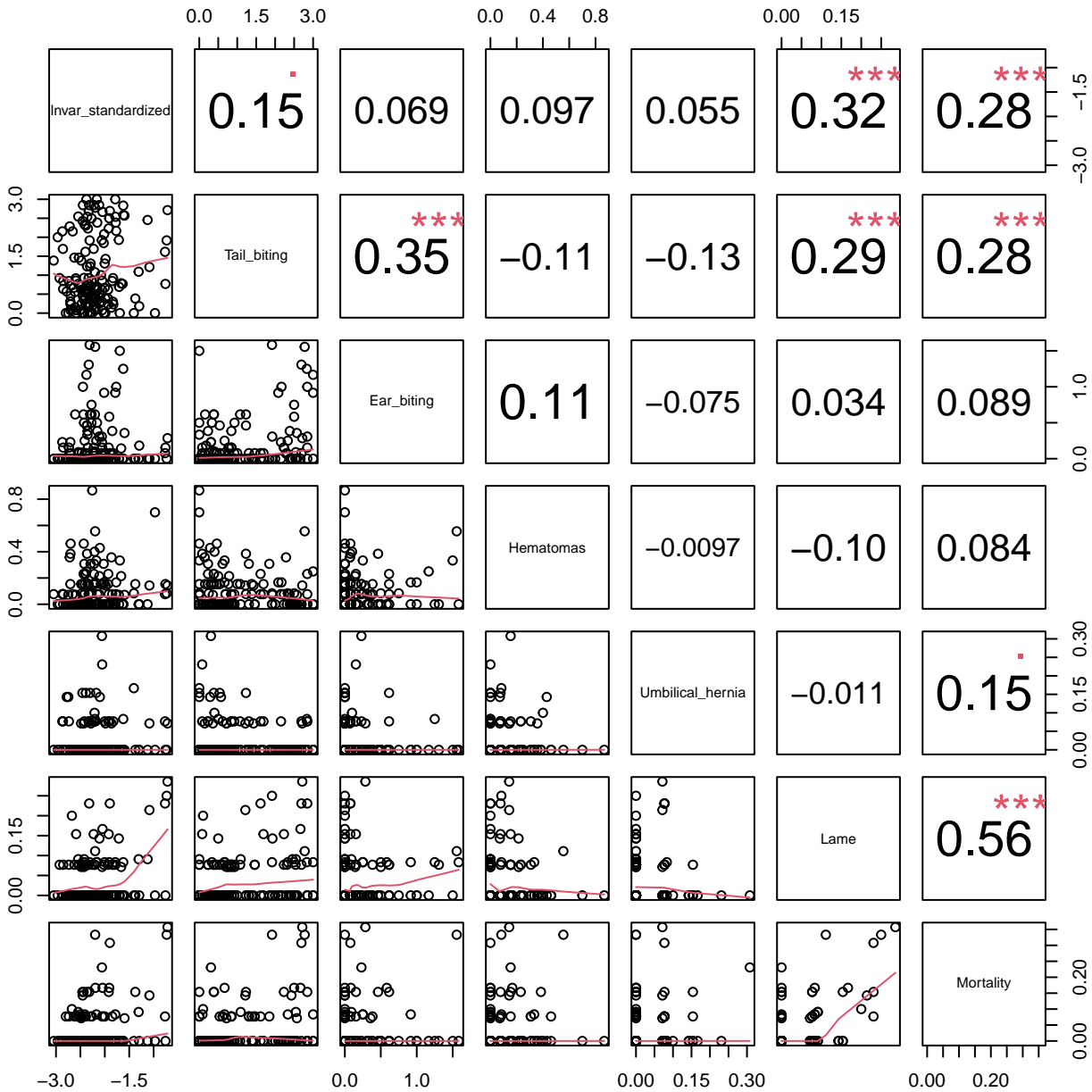

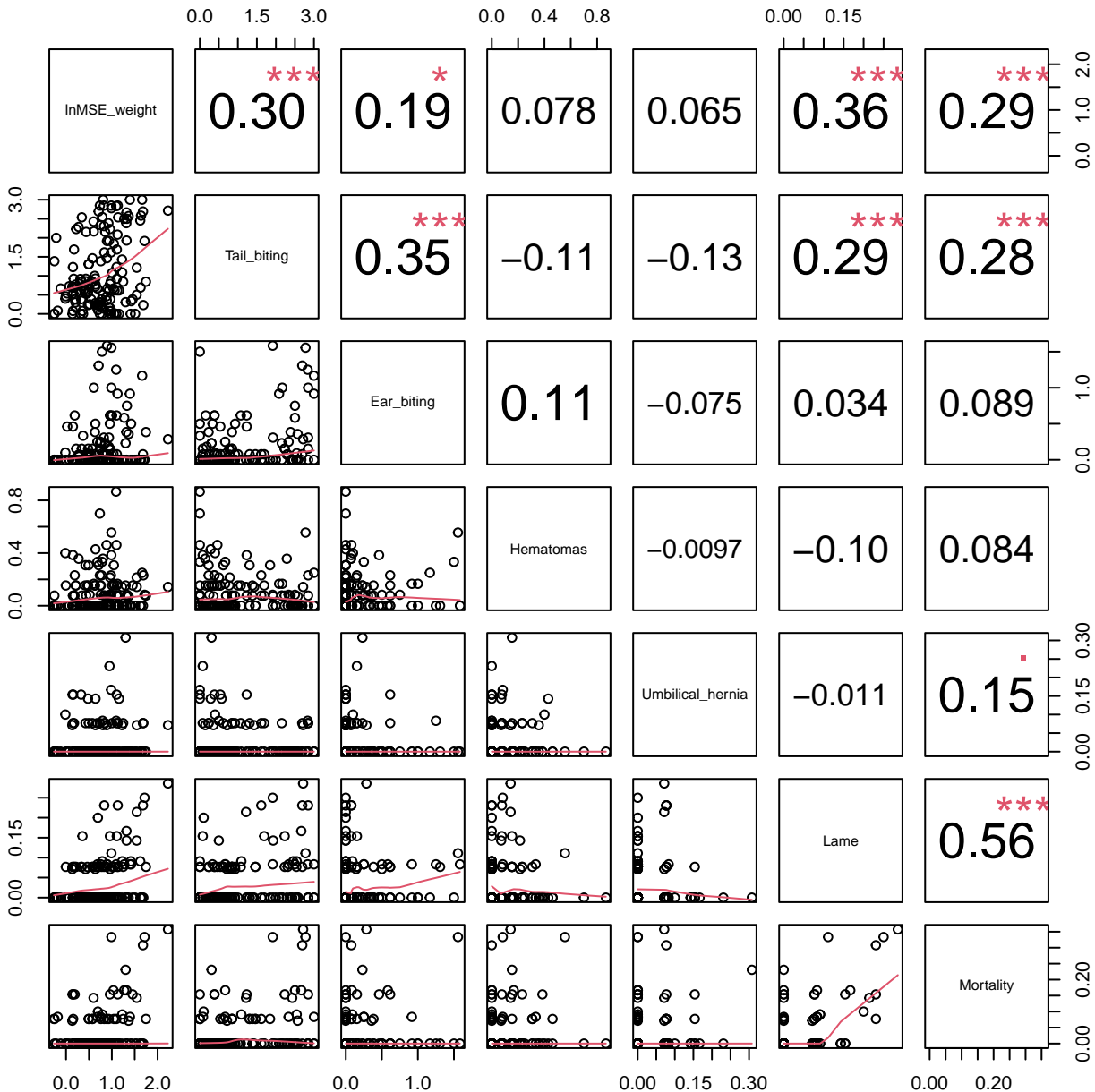

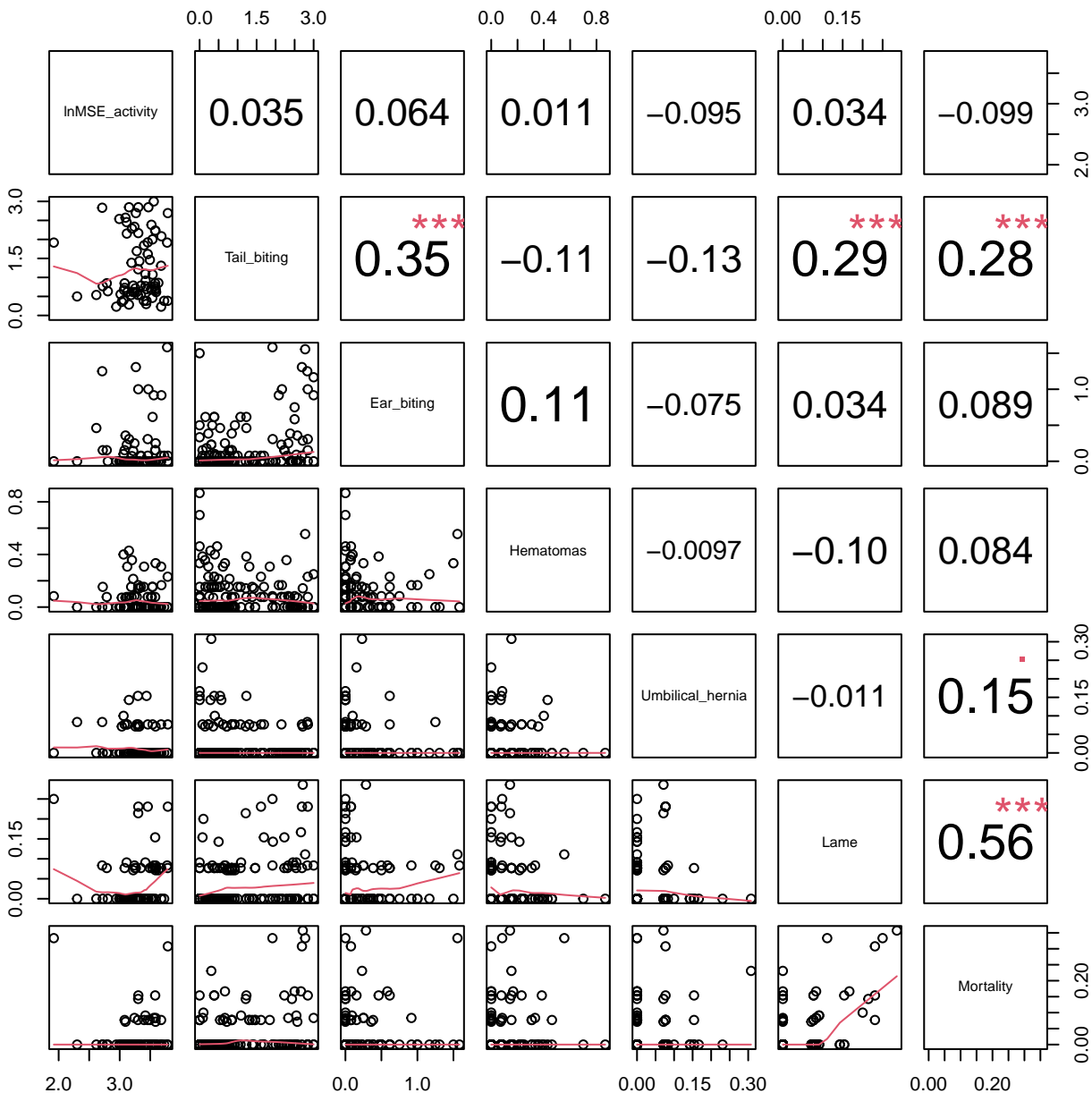

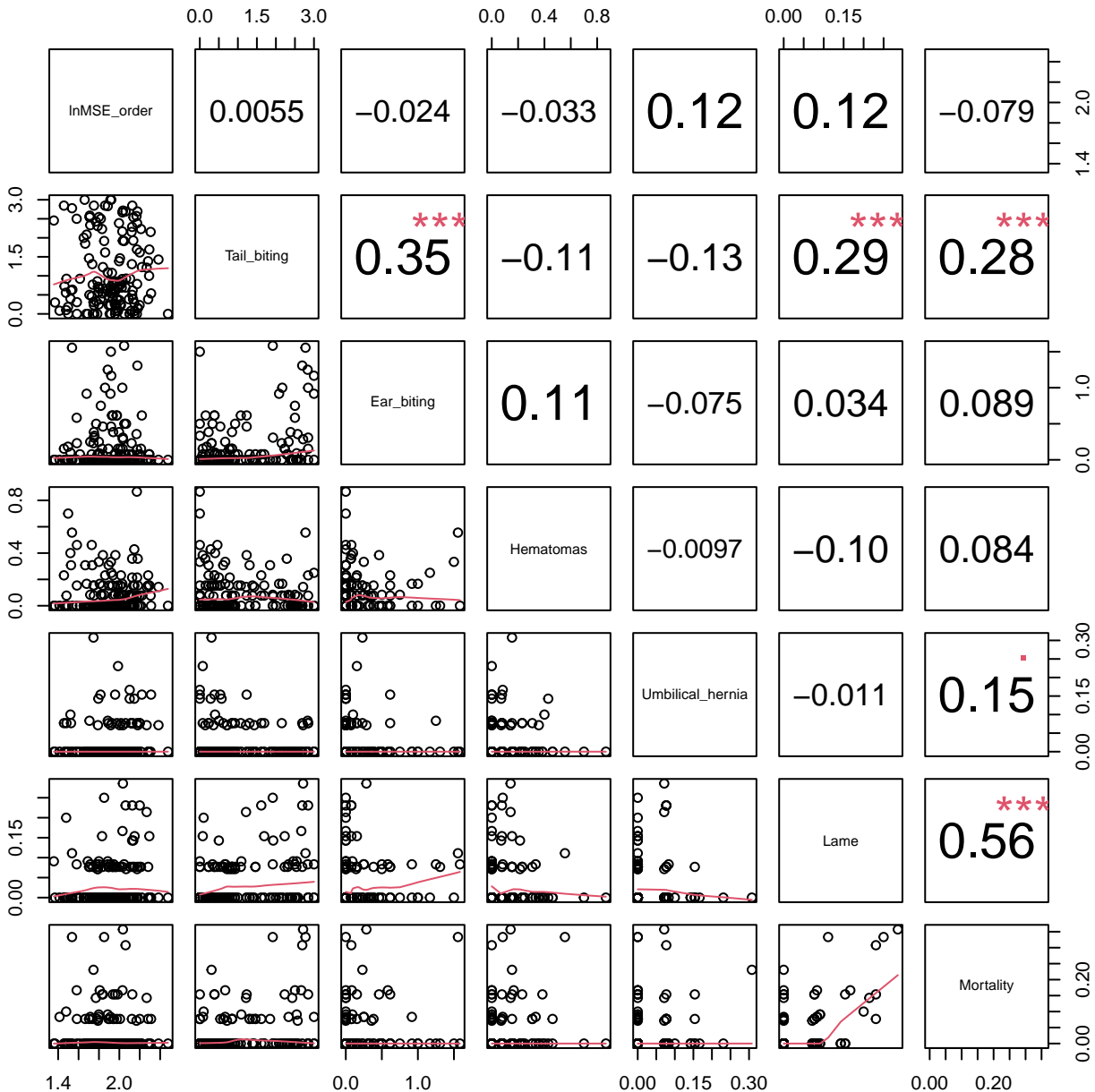

Supplement: Supplementary file 7 — Additional file 7: Figure S7. Pairwise correlation plots of resilience traits according to physical abnormality scores at the pen level. Pairwise correlation plots for all evaluated resilience traits according to mortality and physical abnormality scores, based on the mean score at the pen level. Below the diagonal the pairwise correlation plots are shown. Above the diagonal Pearson correlations are shown. °: correlation is significantly different from zero with p < 0.10. *: correlation is significantly different from zero with p < 0.05. **: correlation is significantly different from zero with p < 0.01. ***: correlation is significantly different from zero with p < 0.001. [file 12711_2024_919_MOESM7_ESM.pdf]

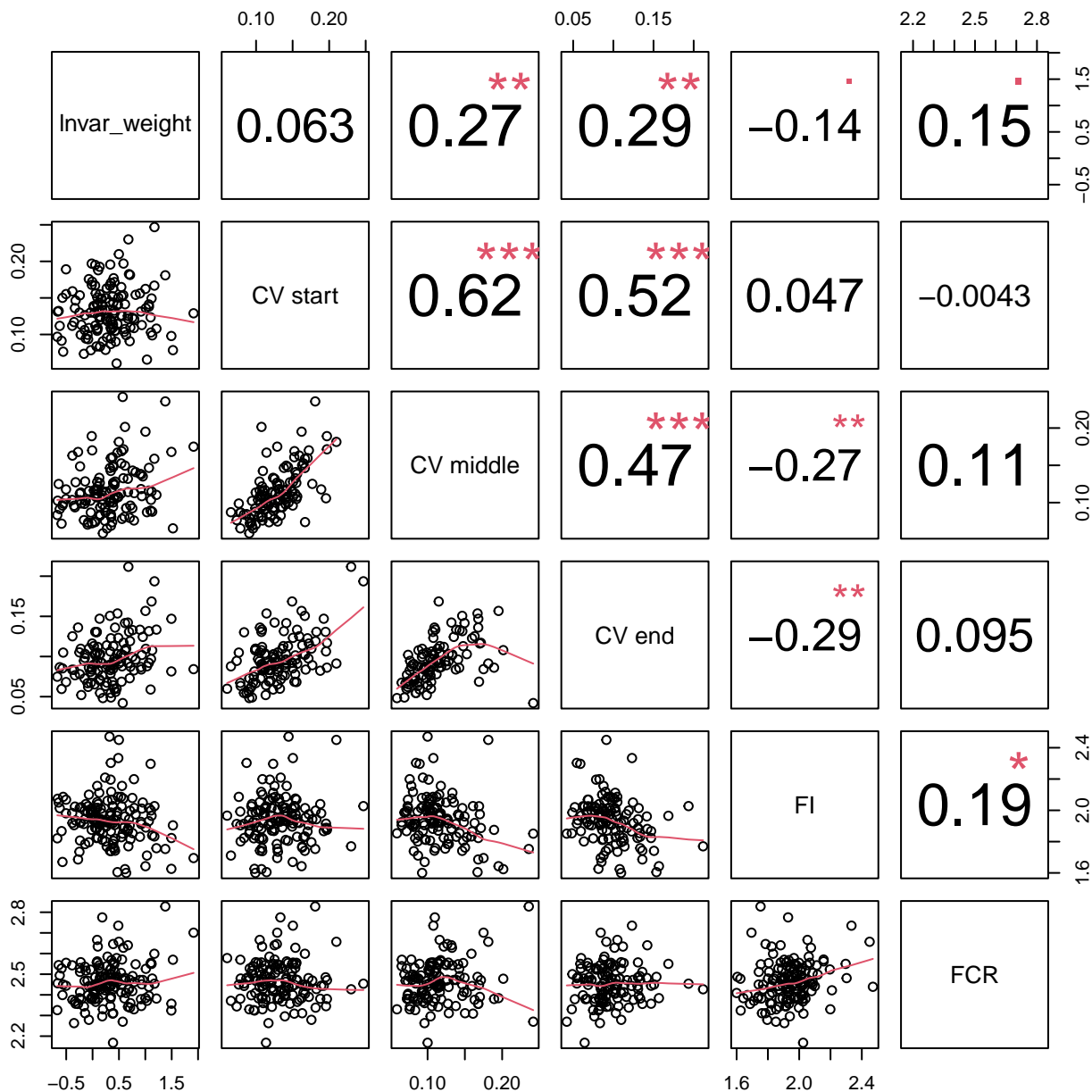

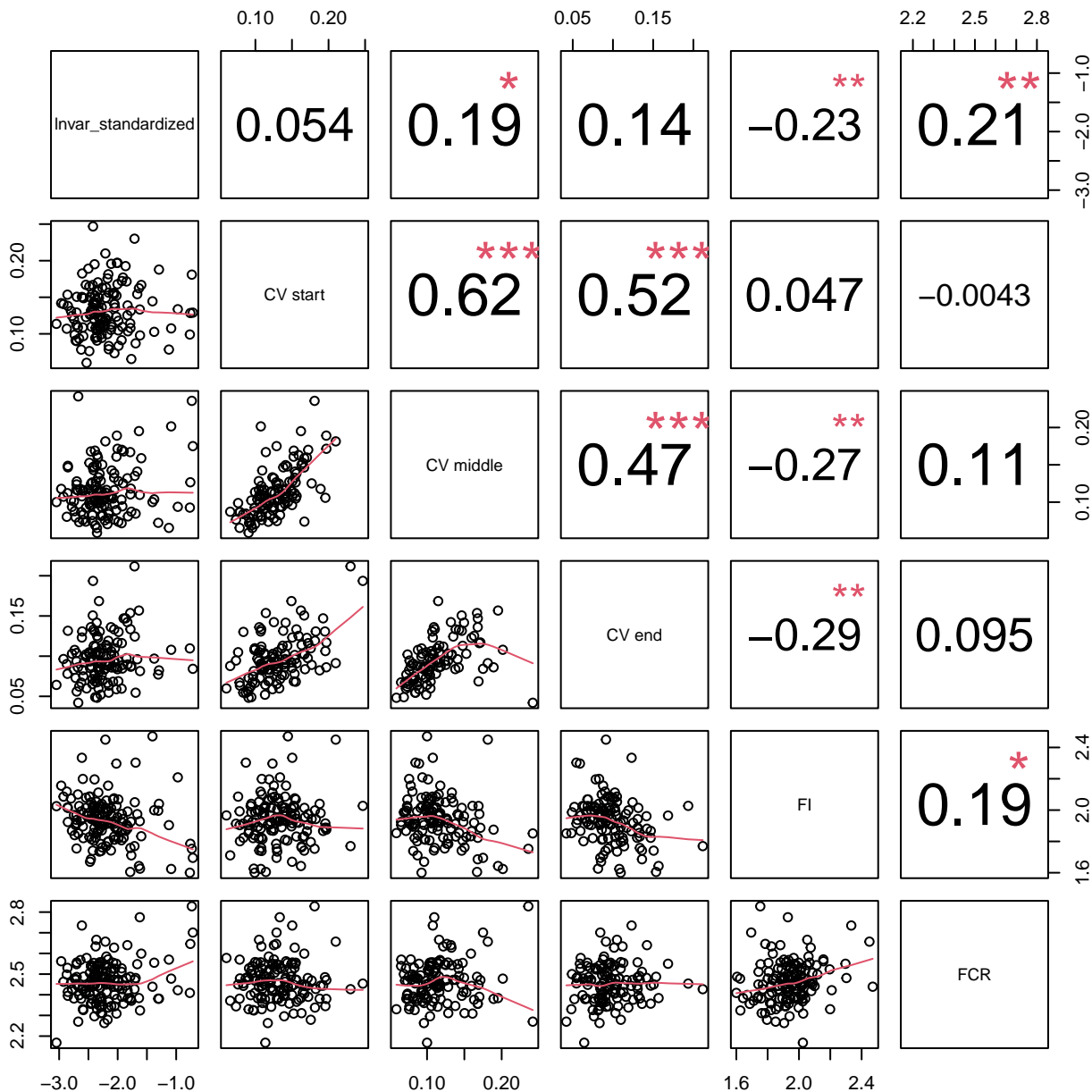

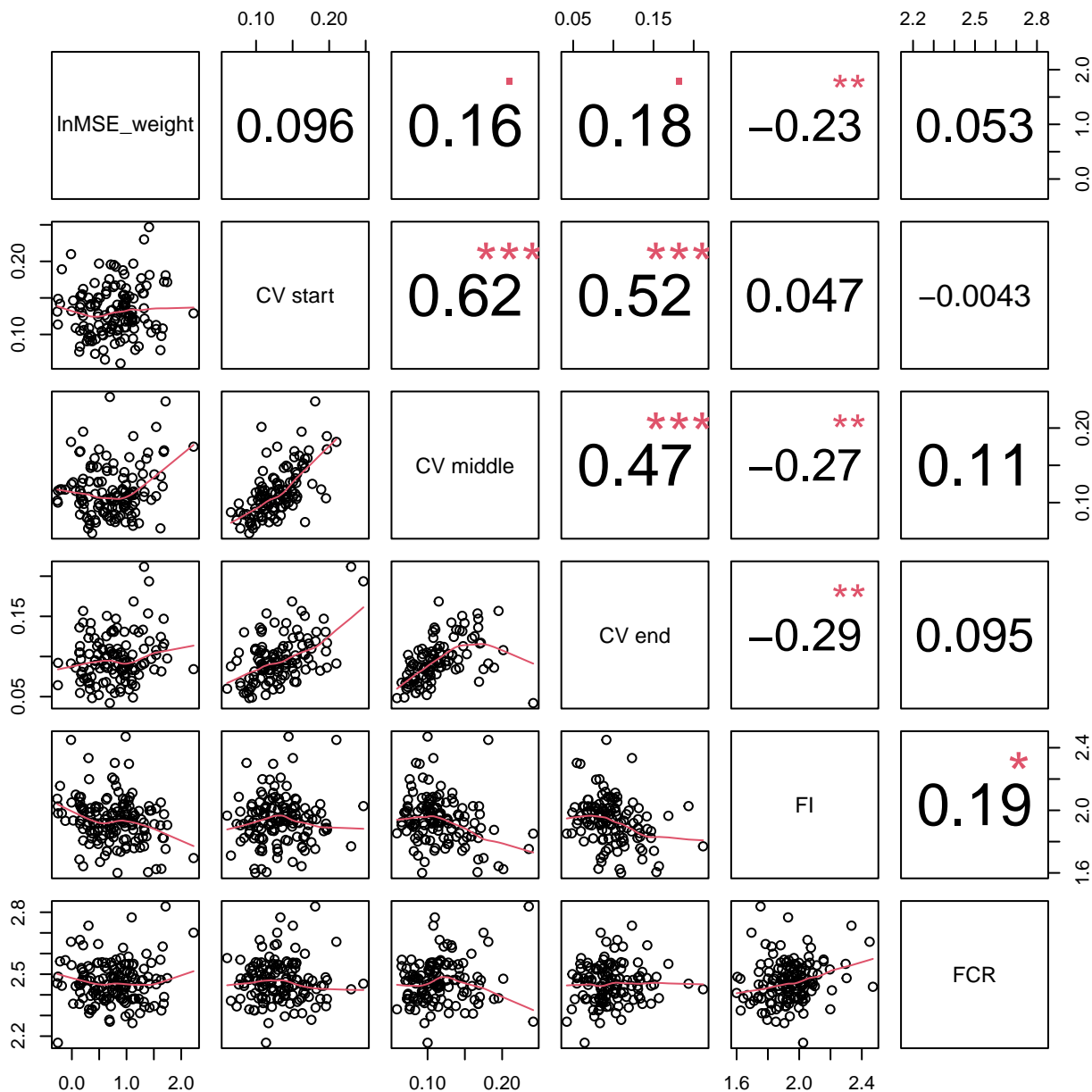

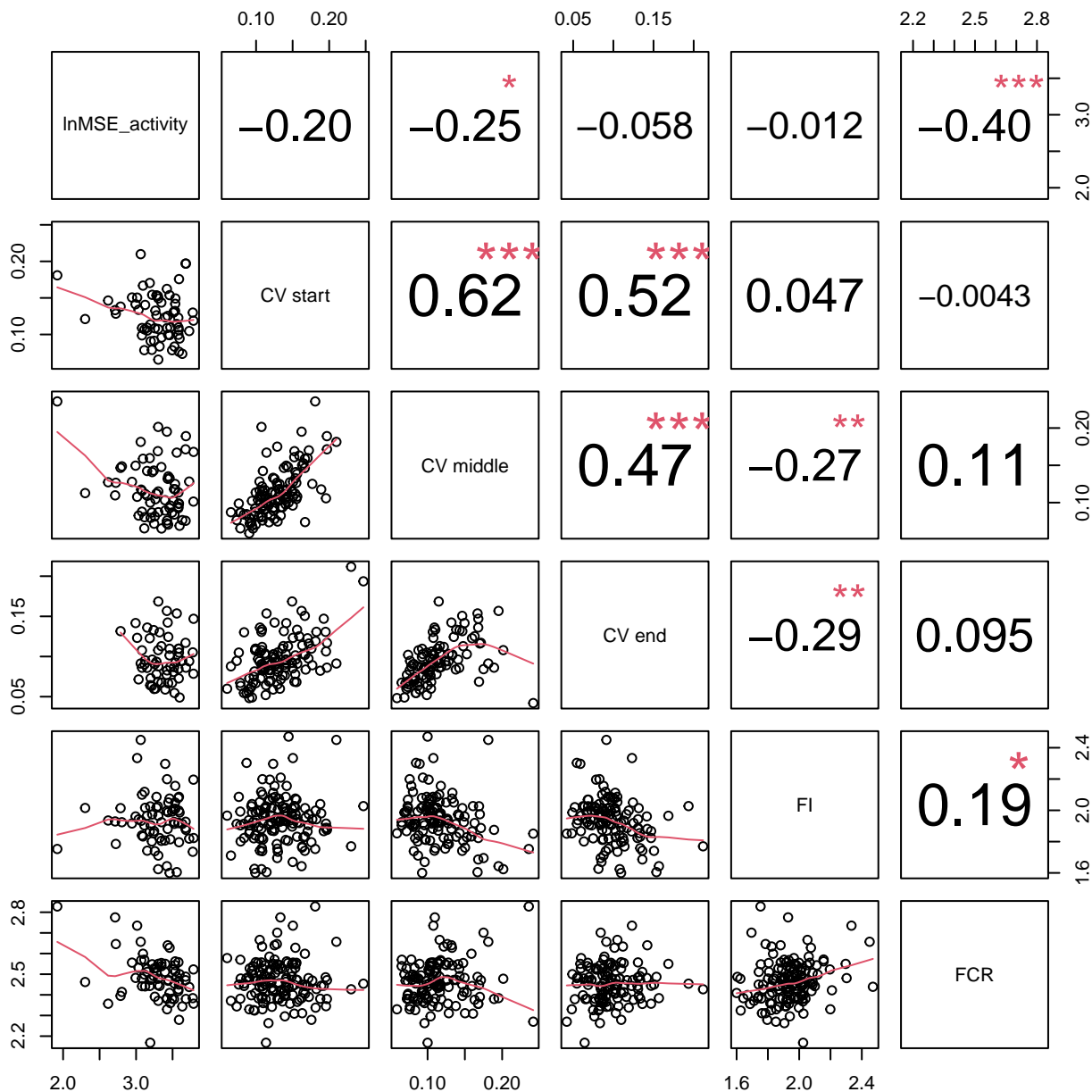

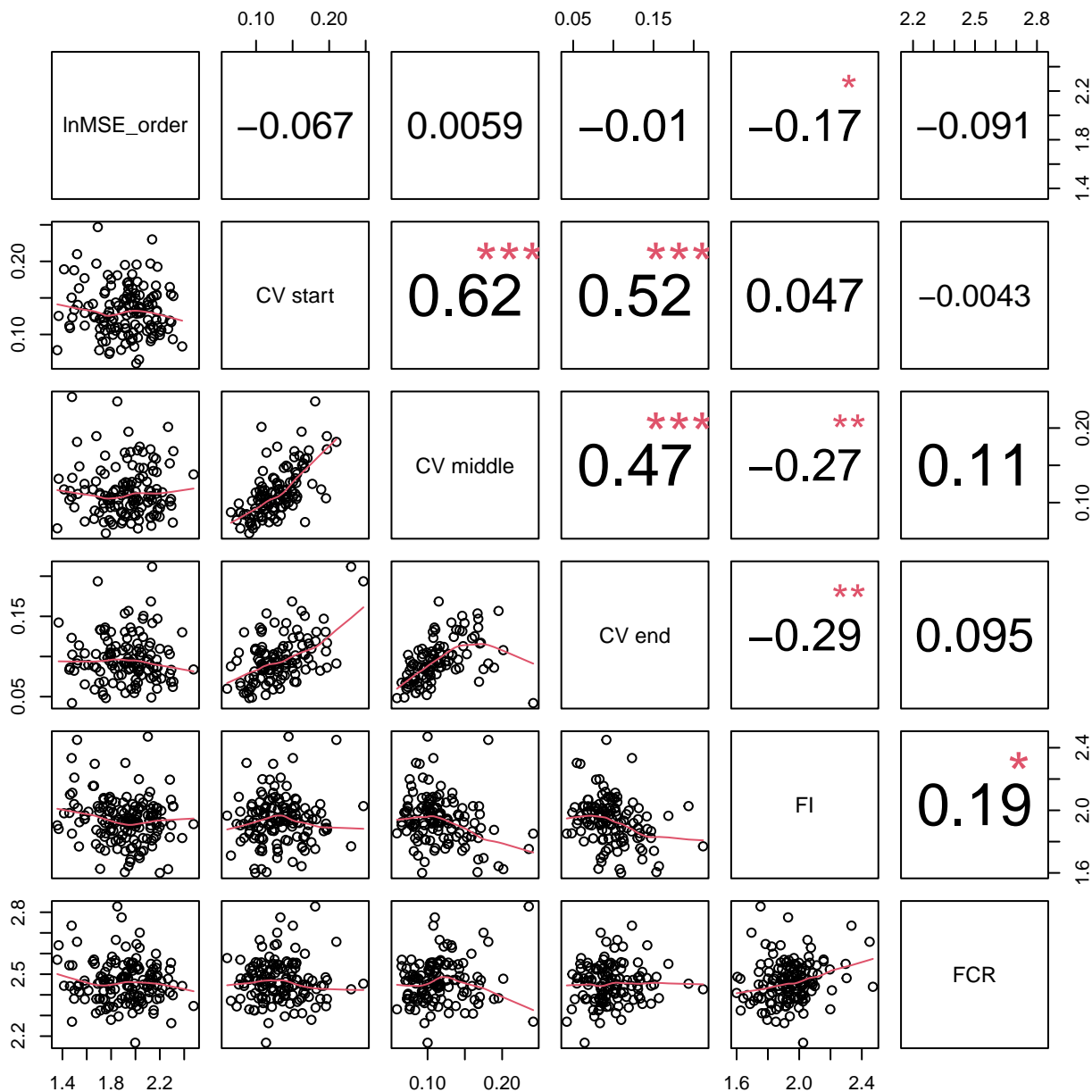

Supplement: Supplementary file 8 — Additional file 8: Figure S8. Pairwise correlation plots of resilience traits according to uniformity, feed intake and feed conversion ratio at the pen level. Pairwise correlation plots for all evaluated resilience traits according to coefficient of variation (CV) at the start, middle and end of the finishing period, as well as the feed conversion ratio. As these traits were recorded at the pen level, the pen-mean of the resilience trait was calculated. Below the diagonal the pairwise correlation plots are shown. Above the diagonal Pearson correlations are shown. °: correlation is significantly different from zero with p < 0.10. *: correlation is significantly different from zero with p < 0.05. **: correlation is significantly different from zero with p < 0.01. ***: correlation is significantly different from zero with p < 0.001. [file 12711_2024_919_MOESM8_ESM.pdf]

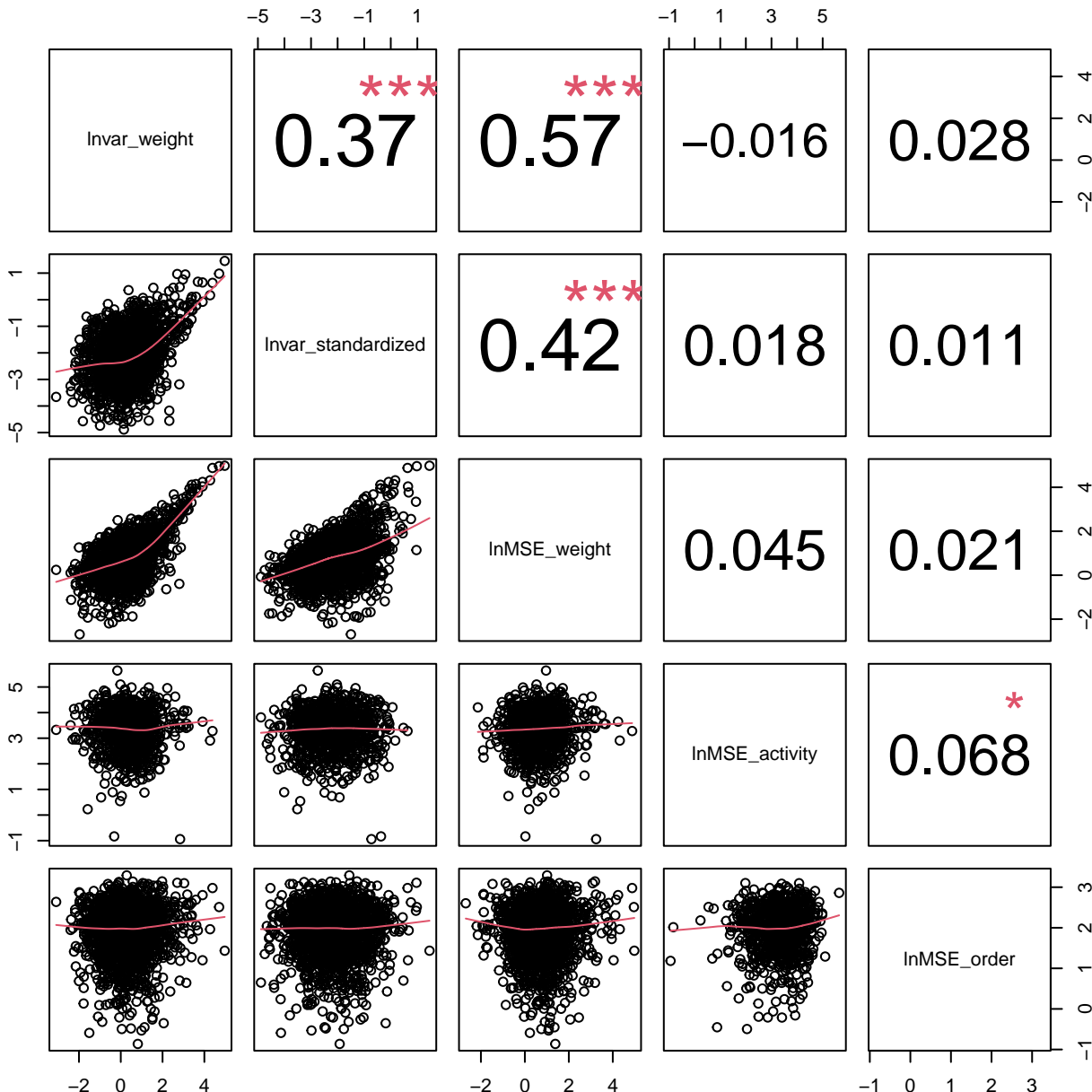

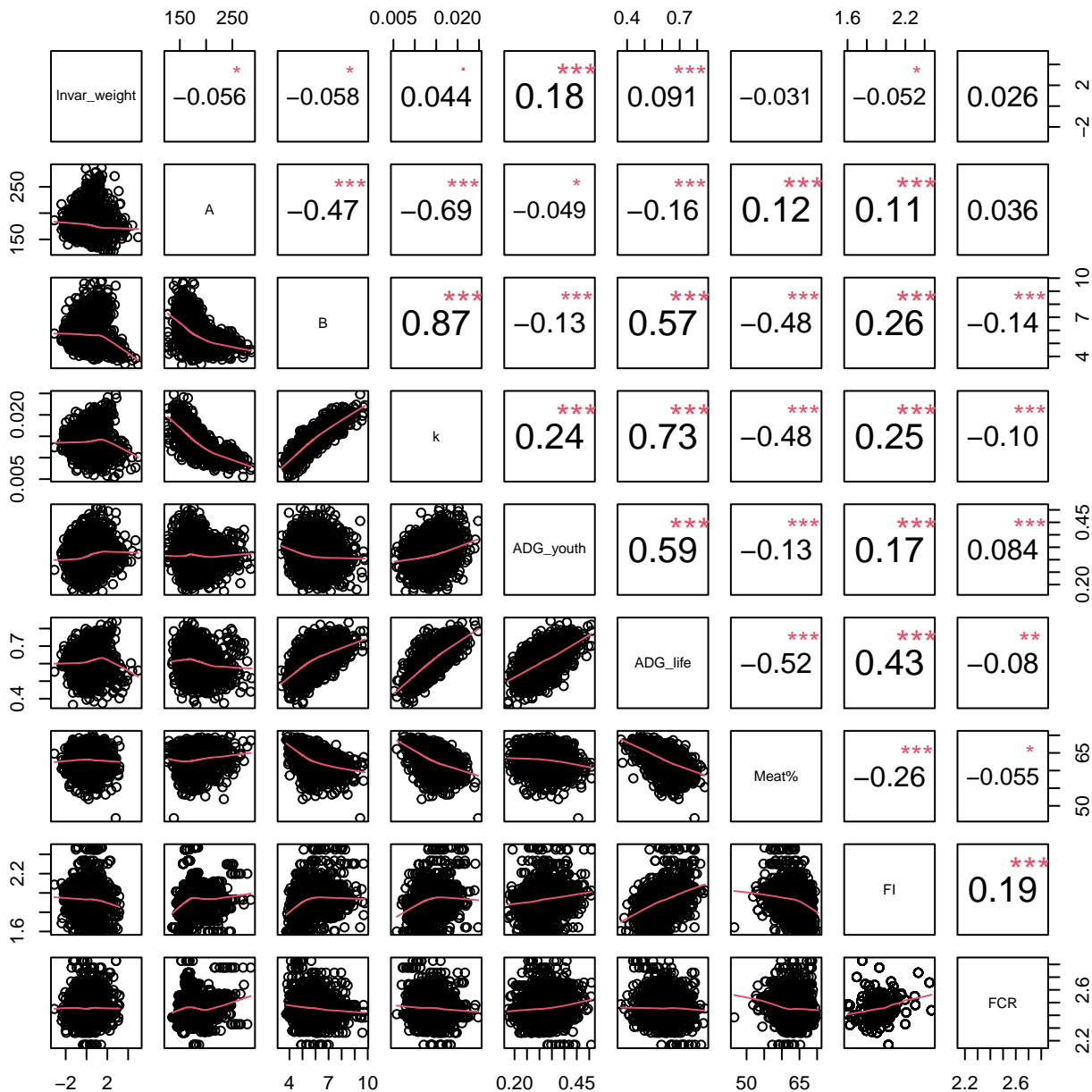

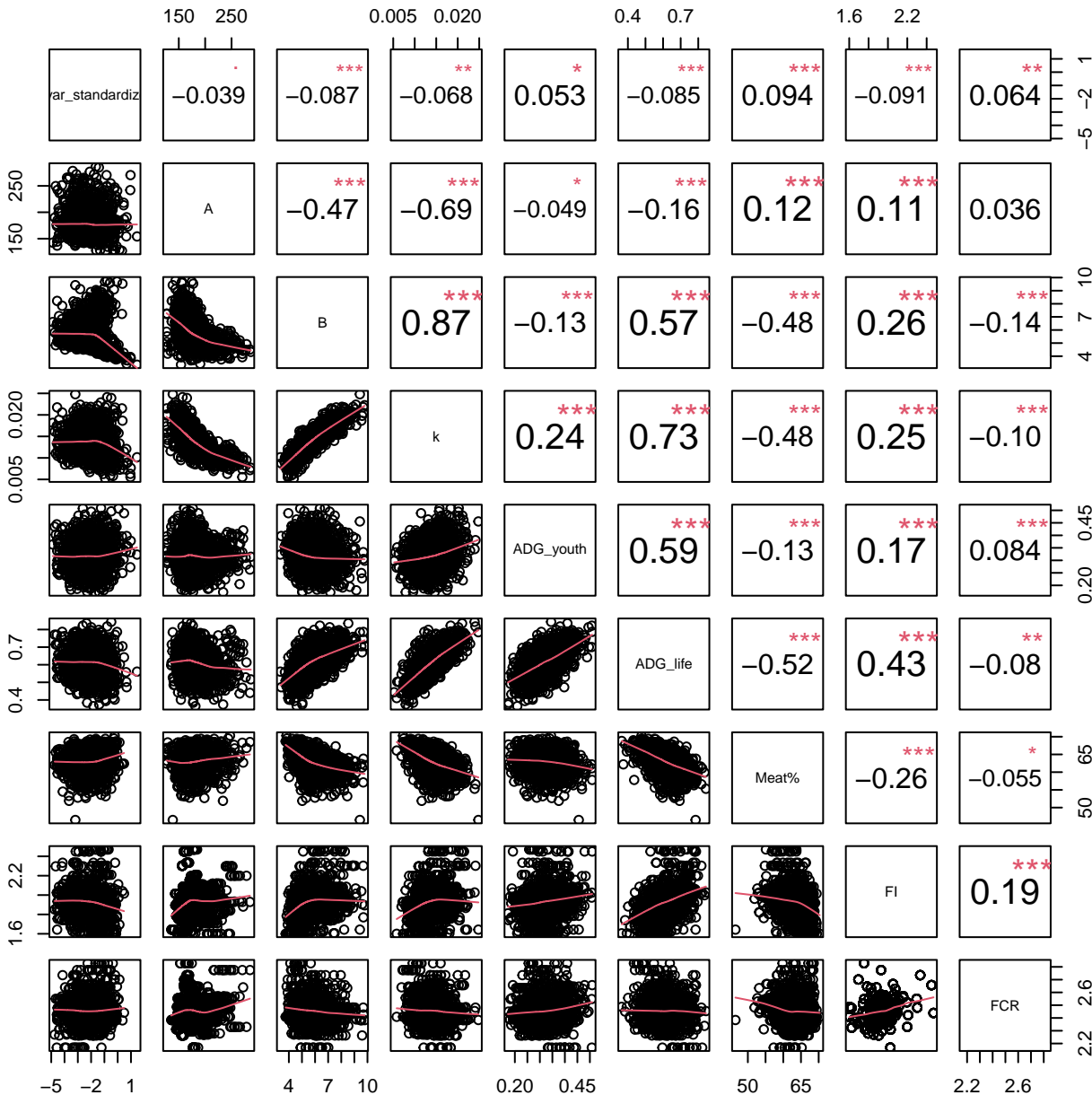

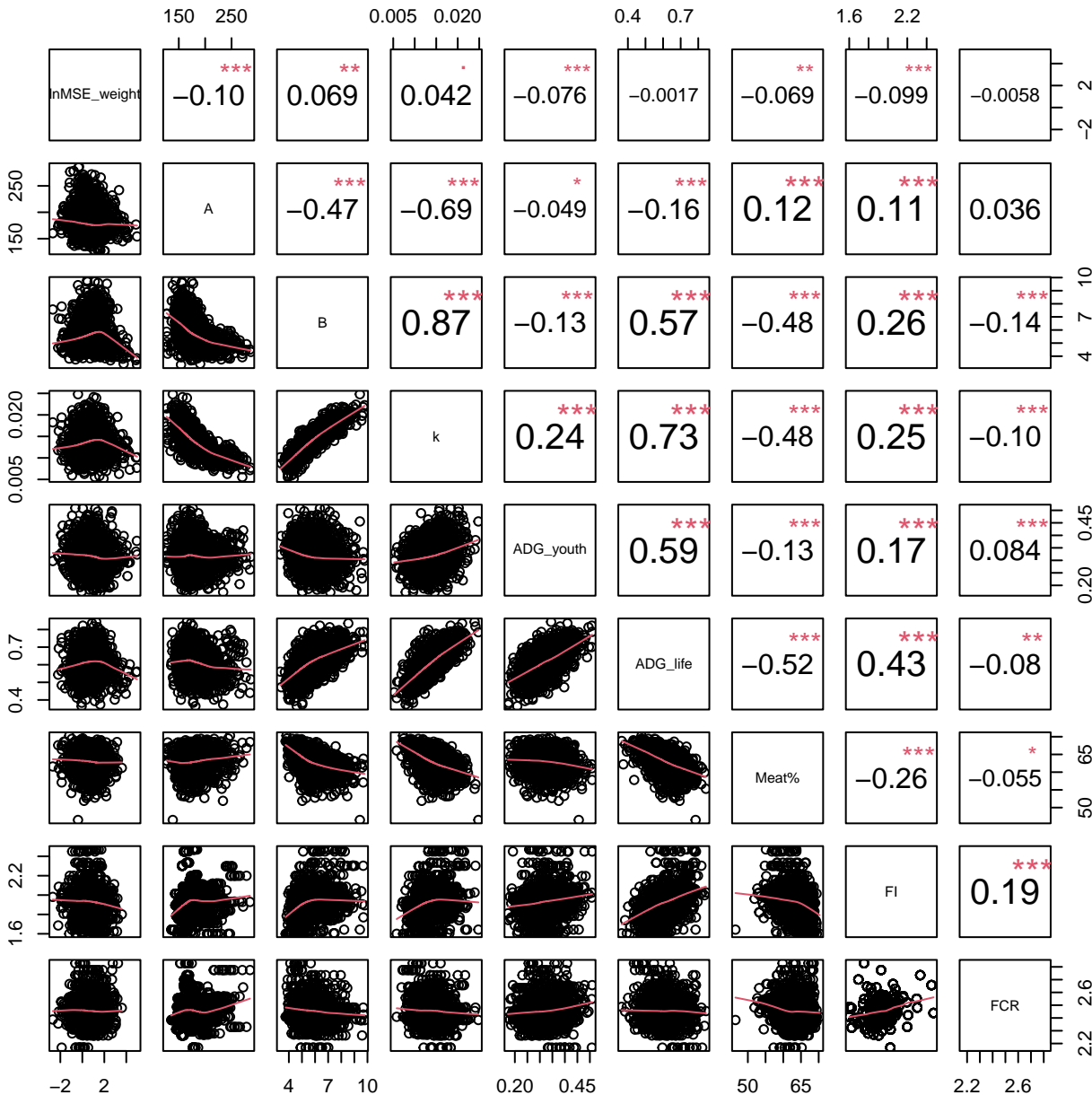

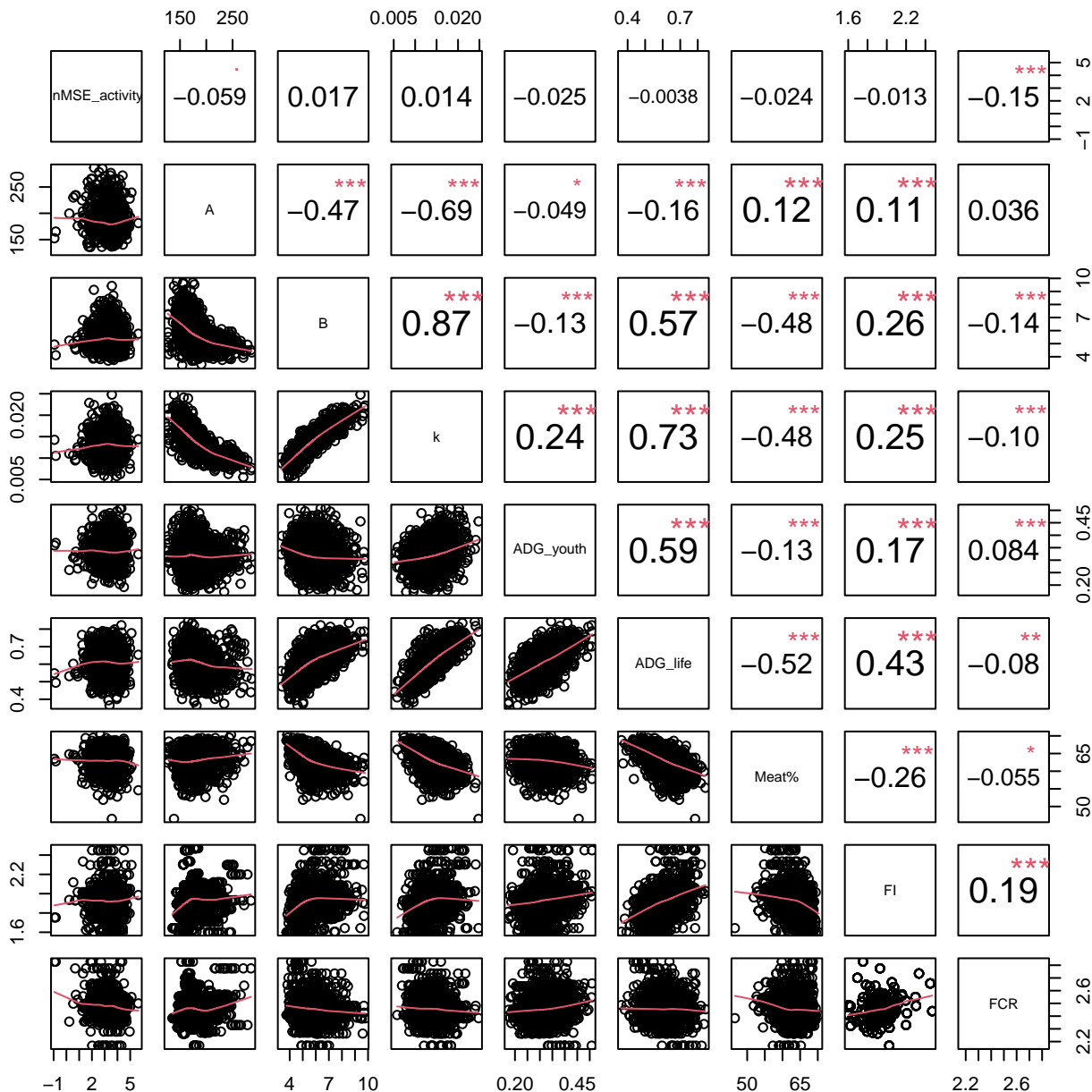

Supplement: Supplementary file 9 — Additional file 9: Figure S9. Pairwise correlation plots of resilience traits according to production traits at the individual level. Pairwise correlation plots within all evaluated resilience traits and between resilience traits and production traits, such as the Gompertz growth curve parameters (A, B, k), average daily gain at start (ADGyouth) and over the pigs life (ADGlife), the recorded meat percentage at slaughterhouse, mean feed intake at pen level (FI) and mean feed conversion ratio at the pen level (FCR). Note that the correlations with FI and FCR might be inflated here, as these were recorded at the pen level, but correlated with traits recorded at the individual level. The correlations at the pen level, without possible inflation of the correlation were also constructed (see Additional file 8 Figure S8). Below the diagonal the pairwise correlation plots are shown. Above the diagonal Pearson correlations are shown. °: correlation is significantly different from zero with p < 0.10. *: correlation is significantly different from zero with p < 0.05. **: correlation is significantly different from zero with p < 0.01. ***: correlation is significantly different from zero with p < 0.001. [file 12711_2024_919_MOESM9_ESM.pdf]
